# Reliability of 3D Lower Extremity Movement Analysis by Means of Inertial Sensor Technology during Transitional Tasks

**DOI:** 10.3390/s18082638

**Published:** 2018-08-11

**Authors:** Rob van der Straaten, Annick Timmermans, Amber K. B. D. Bruijnes, Benedicte Vanwanseele, Ilse Jonkers, Liesbet De Baets

**Affiliations:** 1 REVAL Rehabilitation Research Center, Hasselt University, Agoralaan building A, 3560 Diepenbeek, Belgium; annick.timmermans@uhasselt.be (A.T.); liesbet.debaets@uhasselt.be (L.D.B.); 2 Department of Orthopaedics, Ziekenhuis Oost-Limburg, Schiepse Bos 6, 3600 Genk, Belgium; amber.bruijnes@zol.be; 3 Department of Movement Sciences, Human Movement Biomechanics, KU Leuven, Tervuursevest 101, 3001 Leuven, Belgium; benedicte.vanwanseele@kuleuven.be (B.V.); ilse.jonkers@kuleuven.be (I.J.)

**Keywords:** inertial sensors, motion analysis, biomechanics, repeatability, functional movement

## Abstract

This study assesses the reliability and agreement of trunk and lower limb joints’ 3D kinematics, measured by inertial measurement units, during walking and more demanding movement tasks. For data analysis, tasks were divided in open and closed chain phases. Twenty healthy participants were included. On day one, measurements were conducted by “Operator 1” and “Operator 2” to determine between-operator reliability/agreement. On day two, the measurements were conducted by Operator 1, in order to determine within-session reliability/agreement. Furthermore, between-session reliability/agreement was assessed based on data from Operator 1, captured on day one and two. Within-session reliability/agreement was high, and better than between-session and between-operator results for all tasks. The results for walking were generally better than for other movement tasks, for all joint kinematics, and for both open and closed chain phases. Only for the ab/adduction and flexion/extension angles during forward and sideward lunge, reliability and agreement results were comparable to walking, for both the open and closed chain phases. The fact that lunges show similar reliability results than walking for open and closed chain phases, but require more motor control to perform, indicates that the performance of lunges might be interesting to use in further research aiming to identify kinematic differences between populations.

## 1. Introduction

Inertial sensor motion technology provides new opportunities for human motion analysis. This technology combines signals from accelerometers, gyroscopes, and magnetometers to determine the orientation and position of a body segment [1,2]. Based on the orientation and position of multiple body segments, joint kinematics are subsequently calculated in three dimensions (3D) [3]. Although inertial sensor systems are promising for use in clinical settings, that is, they are mobile and relatively inexpensive, there are also drawbacks related to the use of inertial sensor technologies. Accurate placement and strapping of the inertial sensor are essential to reduce soft tissue artefacts [4,5] and the proximity of ferromagnetic materials should furthermore be avoided because this disturbs the determination of the orientation and position of a body segment [3]. Before further introducing these systems into clinical practice, it is thus of utmost importance to establish the validity and reliability of these systems.

Based on a recent systematic review, it has been shown that there are multiple studies that investigated the validity or reliability of (3D) kinematics measured by an inertial sensor system [6]. The majority of these validity or reliability studies only included the assessment of knee joint angles and only assessed the kinematics during level walking. Regarding the validity, the flexion/extension angles showed the highest accuracy, in comparison to the ab/adduction angles and the in/external rotation angles. With regard to the reliability, few studies investigated the reliability and agreement of the lower limb joints’ (i.e., hip, knee, and ankle) kinematics measured by an inertial sensor system [7,8,9]. Cloete and Scheffer reported good reliability using the coefficient of multiple determination (CMD) and the coefficient of multiple correlation (CMC) for within-day (CMD 0.786–0.984; CMC 0.881–0.992) and between-day reliability (CMD: 0.771–0.991; CMC 0.872–0.995) for 3D lower limb joint kinematics during level walking [8]. Neüsh et al. [9] reported similar within-day reliability and agreement results for sagittal plane joint kinematics during walking (CMC > 0.95) and running (CMC > 0.90), with measurement errors < 5° and < 7°, respectively. The most recent reliability study of Al-Amri et al. [7] also included, besides walking, a squat and a vertical jump. Acceptable within-operator (between-day) and between-operator reliability and agreement of 3D lower limb joint kinematics during walking and squatting were reported, with intraclass correlations (ICCs) higher than 0.6. Only for hip flexion/extension, knee internal/external rotation and ankle abduction/adduction and supination/pronation, lower ICCs were found. The standard error of the measurement (SEM) was acceptable (<5°) for all joints and rotations during walking and squatting. The reliability of the joint kinematics during the vertical jump was substantially lower, especially for the between-operator reliability [7].

In the aforementioned studies, the reliability is tested in tasks that are mainly relevant when used with healthy individuals or athletes. These tasks are, however, not appropriate for or feasible to perform in other populations, such as persons with degenerative joint diseases. As it is assumed that (mal)adaptive strategies are more prominent during physically demanding and challenging tasks [10], it might be beneficial to add forward and sideward lunges and stair ascending/descending to a movement protocol for the assessment of (mal)adaptive movement strategies in persons with degenerative joint pathology. Moreover, these studies assessed the reliability of lower limb joint kinematics, without including trunk kinematics. However, given the multidimensionality of the locomotor system, movements can be achieved by multiple musculoskeletal strategies. In the case of functional limitations, because of pain or pathology (e.g., cartilage damage in osteoarthritis), an individual might show (mal)adaptive movement strategies in order to be able to perform specific movements. For example, based on optoelectronic measurements, it has been shown that lateral trunk lean is an effective strategy to unload the knee joint in persons with knee osteoarthritis [11]. More specifically, these authors reported that lateral trunk lean during the early stance phase was more effective in unloading the knee as lateral trunk lean during late stance [11]. Therefore, in addition to the assessment of the lower limb joints (based on inertial sensors), it might be of added value to assess trunk kinematics during distinct phases of the movement task (e.g., swing and stance phase).

Given the fact that inertial sensor based motion analysis relies on a proper use of the system and the fact that the reliability and agreement are task-specific, it is essential to assess the reliability and agreement of any newly developed movement protocol, before it can be used for clinical reasoning or to evaluate treatment efficacy. Therefore, in this study, a movement protocol was developed, combining trunk and lower limb joint kinematic measurements, assessed during functional and physically demanding tasks for persons with degenerative hip and knee problems. This way, the protocol can be used in future studies on the evaluation of (mal)adaptive movement strategies in persons with hip or knee osteoarthritis. The aim of this study is to specifically assess the within-session, between-session, and between-operator reliability and agreement of the physically demanding tasks and discuss these results in light of the reliability and agreement results of a standard gait analysis. Furthermore, reliability and agreement results of the different phases of the movement tasks will be discussed in order to make recommendations for parameters selection for future studies.

## 2. Materials and Methods

### 2.1. Participants

Twenty healthy volunteers, who were selected from a local network of seniors and relatives, participated in the study. For inclusion in the study, healthy participants had to be 50–75 years old, able to walk 10 m, able to ascent and descent a staircase (i.e., 4 steps), and able to understand the Dutch language. Participants were excluded if they had pain or pathology in the lower limb joints or torso, or had any systemic or neurological disease. Before participation, all participants signed the informed consent, which was approved by the ethical committee of the academic hospital Leuven (reference no. s-59857).

### 2.2. Data Collection

#### 2.2.1. Instrumentation

**Figure 1:**
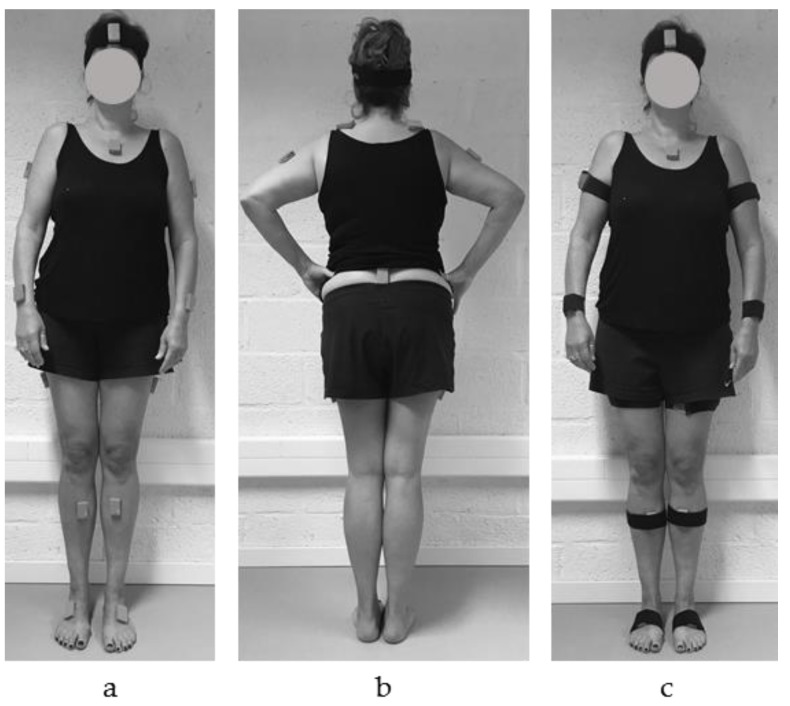
Inertial sensor position in (**a**) anterior and (**b**) posterior view without straps, and (**c**) anterior view with straps to preload the inertial sensor and minimize soft tissue artefacts.

In order to assess the trunk and lower limb joint kinematics, a full body configuration was applied using 15 inertial sensors (MVN BIOMECH Awinda, Xsens Technologies, Enschede, The Netherlands). The inertial sensors (MTw Awinda) were placed according to the guidelines provided in the MVN user manual [12]. On the lower limbs, including the pelvis, the inertial sensors were positioned on the dorsal side of the foot, the medial surface of the tibia (underneath the tibial tuberosity), halfway laterally on the thigh and on L5/S1 (Figure 1a,b). For the upper body, the inertial sensors were positioned on the dorsal side of the forearm (most distal, between ulna and radius), halfway the upper arm slightly dorsal of the middle line, along the superior border of the scapulae, halfway on the flat part of the sternum, and in the middle of the forehead (Figure 1a,b). The inertial sensors were positioned directly on the skin using double-sided adhesive tape, and were secured with a strap in order to preload the inertial sensor and to minimize soft tissue artefacts (Figure 1c) [4,13]. The corresponding MVN BIOMECH Awinda software (MVN Studio 4.4, firmware version 4.3.1) was used to calculate the (full-body) joint kinematics. In this software, it is required to provide the participants’ body dimensions as an input for the full body configuration/model, to scale the body segments [3]. Additionally, in order to align the sensor’s orientation to the segment’s orientation, a static calibration was performed. The static calibration was done while the participant was standing in an N-pose position; standing in an upright neutral position, with the arms along the body, their feet parallel beside each other pointing forward, and making sure that both legs and arms are in a straight line downwards.

#### 2.2.2. Movement Protocol

The movement protocol included four tasks: walking, forward lunge, sideward lunge, and stair ascending and descending. For level walking, the participants walked 10 m at their own comfortable walking speed. The step length of the forward and sideward lunge was standardized to 70% of the participants’ leg length (distance from the greater trochanter to the ground). For the upstairs and downstairs task, the participant ascended and descended the stairs (i.e., step over step). The staircase contained four steps with steps 30-cm deep and 16-cm high. All tasks were executed barefoot and before task execution, the participants were instructed by the operator and the task was practiced to familiarize a uniform task execution, according to the instruction of the operator. A detailed description of the instructions to the participant is provided in Table 1.

Each participant performed the protocol (four tasks, five repetitions per task) three times, on two different days. On day one, the participant executed the protocol twice. In the first session “Operator 1” performed the measurement, that is, measuring body dimensions, positioning of the inertial sensors and straps, performing the static calibration, and instructing the participants before every new task.

After the first session, all inertial sensors were removed and a break of half an hour was implemented in order to rest and allow skin and strap marks to disappear. After the break, “Operator 2” performed the measurement, in which the procedure was repeated. The order in which the operators guided the measurements on day one was randomized. On the second test day, between 5 and 20 days apart, the participant returned to the lab where the entire procedure was repeated by the same operator. The operators, both experienced in conducting motion analysis studies, gave structured and concise instructions to the participants (Table 1) and evaluated the task execution. In any case that the task execution deviated from the instructions given by the operators, an additional trial was recorded.

### 2.3. Data Analysis

Three-dimensional joint angles from the trunk, pelvis, hip, knee, and ankle were directly derived from the MVN software [3]. Within this software, body segments of the anatomical model are defined based on the recommendations of the international society of biomechanics [14], where the *x*-axis represents frontal plane joint movements (abduction/adduction), the *y*-axis the transversal plane joint movements (internal/external rotation), and the *z*-axis the sagittal plane joint movements (flexion/extension). Movement cycles were divided into sub-phases (Table 2), based on the acceleration of the shank sensor and angular velocity of the foot sensor [15,16]. Therefore, a custom written peak detection algorithm in Matlab (2016b, Mathworks, Inc., Natick, MA, USA) was used to define initial foot contact and toe-off. Based on these parameters, the joint angles from trunk, hip, knee, and ankle were normalized from 0–100% for each sub-phase (Table 2). Minimum and maximum joint angles were determined per joint for each sub-phase. Before analysis, the first trial was deleted as it can be disturbed by initiation strategies. All trials were visually inspected for incorrect data resulting from technical errors. Only data from the right limb were used for analysis.

### 2.4. Statistical Analysis and Data Interpretation

#### 2.4.1. Statistical Analysis

Statistical analysis was performed using SPSS (version 25, IBM Corporation, Amonk, NY). Reliability of the joint angles was determined based on the intraclass correlation coefficient (ICC), including the 95% confidence interval. Agreement was evaluated by using the standard error of the measurement (SEM), based on the square root of the mean square error term of the analysis of variance (ANOVA) and the minimum detectable change (MDC) between two sessions, using the SEM (MDC = SEM × 1.96 × √2) [17]. Single data was used to calculate the within-session (intra-operator) reliability (ICC2,1) and agreement. Averaged data from four repetitions from two days was used to calculate the between-session (intra-operator) reliability (ICC2,k) and agreement. Averaged data from four repetitions from two operators was used to calculate the between-operator (inter-operator) reliability (ICC2,k) and agreement. ICCs ≥ 0.90 were considered as excellent, 0.70–0.89 good, 0.69–0.40 acceptable, and <0.40 as low [18].

#### 2.4.2. Data Interpretation and Presentation

The within-session reliability and agreement provides information on the natural variability in movement for a given task or phase, that is, repeated movements that are executed in a variable way lead to low within-session ICCs. Between-session and between-operator reliability and agreement data on the other hand provides information about the degree of variation that exists between sessions (i.e., operator dependent sources of variation such as task explanation or sensor placement/calibration, or participant dependent sources of variation). If the source of variation is operator dependent, better between-session than between-operator reliability and agreement results are expected across all tasks. If between-session and between-operator reliability and agreement are equal, but worse than within-session reliability and agreement, natural variability in task execution between different test sessions is assumed. Furthermore, it should be taken into account that the magnitude of the ICC is dependent on between-subject variability [17]. High ICCs can hide poor trial-to-trial consistency in the case of high between-subject variability. Conversely, limited between-subject variability could result in poor ICCs even when trial-to-trial consistency is high. Therefore, reliability (i.e., relative consistency) should always be interpreted together with measures of agreement such as SEM and MDC (i.e., absolute consistency). These measures are presented in the same units as the measurements of interest (degrees (°)), and therefore they are interpretable in clinical practice.

To clearly visualize ICCs of multiple angles (trunk, hip, knee, and ankle) and rotations (frontal, transverse, and sagittal), the ICCs are presented in boxplots. One boxplot represents the ICCs from minimum and maximum angles of all movement phases. The bottom of the box and top of the box represent the 25th and 75th percentile, respectively; the band inside the box is the median ICC; and the lower and upper whisker represent the minimum and maximum ICCs, respectively. In addition to the median, the mean ICC of all sub-phases is presented in the boxplot by means of a cross.

## 3. Results

### 3.1. Participants

Twenty healthy participants, 9 male and 11 females with an average age (SD) of 62.7 (8.5) years and body mass index of 24.4 (3.6) kg/m^2^ were enrolled in the study.

### 3.2. Reliability and Agreement

The within-session reliability and agreement are first assessed for each task, phase, and rotation. In the case of good within-session reliability and agreement results, between-session and between-operator reliability and agreement are subsequently reported for these individual tasks, phases and rotations. Individual results (i.e., ICC, SEM, and MDC) of the within-session, between-session, and between-operator are provided in the Supplementary Materials (Tables S1–S5). In addition, waveforms for each task, phase, and rotation are presented in the Supplementary Materials (Figures S6–S10) and a side-by-side visual comparison of the within-session, between-session, and between-operator reliability (ICCs) levels per rotation for all tasks are presented in the Supplementary Materials (Figure S11).

#### 3.2.1. Within-Session Reliability

**Figure 2:**
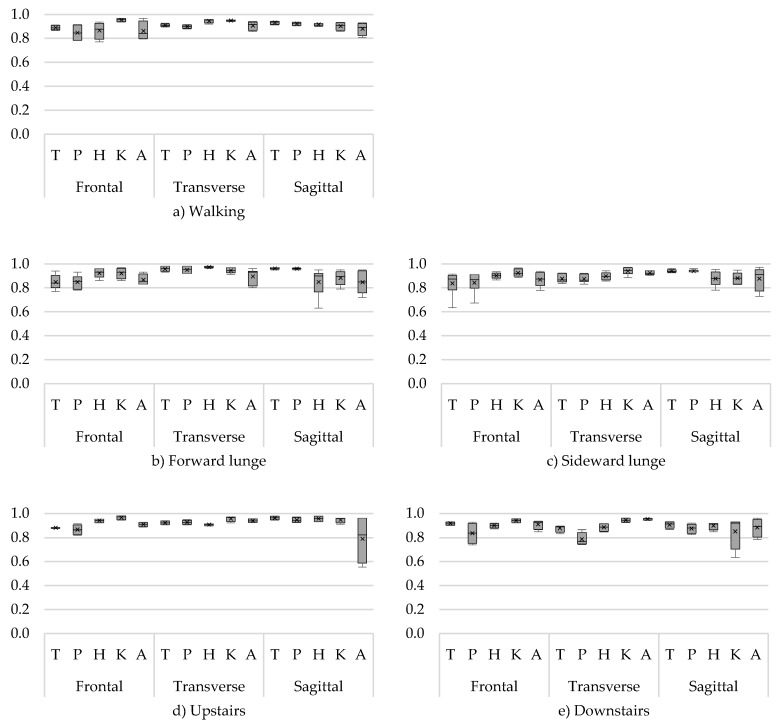
Within-session intraclass correlations (ICCs) of the angles for (**a**) walking, (**b**) forward lunge, (**c**) sideward lunge, (**d**) upstairs, and (**e**) downstairs. With on the *x*-axis the trunk (T), pelvis (P), hip (H), knee (K), and ankle (A) angles for the frontal (F), transverse (T), and sagittal (S) plane, and the ICC on the *y*-axis.

Good to excellent ICCs were found for all joint rotations in all tasks and phases (Figure 2). Walking showed the highest within-session reliability (ICC ≥ 0.77). Good results (ICC > 0.70) were also found for the swing towards foot contact (SWon) and foot contact on the ground (FTon) phases of both the forward and sideward lunge (Figure 2). Only during the swing backwards (SWoff) phase, slightly lower, but still acceptable, ICCs were found for the minimum hip flexion/extension angle (ICC 0.63) of the forward lunge and minimum trunk ab/adduction angle (ICC 0.63) of the sideward lunge. For upstairs and downstairs, good ICCs were found (Figure 2), except for the minimum ankle flexion/extension angle (ICC 0.55) during stance and swing phase and maximum knee flexion/extension angle (ICC 0.63) during the stance phase, respectively.

#### 3.2.2. Between-Session and Between-Operator Reliability

##### Walking

**Figure 3:**
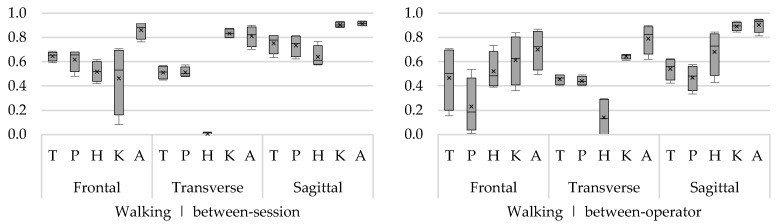
Between-session (**left**) and between-operator (**right**) reliability of minimum and maximum trunk (T), pelvis (P), hip (H), knee (K), and ankle (A) angles during walking in the frontal, transverse, and sagittal plane. The *y*-axis represents the ICC.

The highest between-session ICCs were found for the flexion/extension angles during both the swing and the stance phase of walking (ICC 0.57–0.94) (Figure 3). Between-session ICCs of the ab/adduction angles during the swing and the stance phase were slightly lower but still ranged from acceptable to excellent (ICC 0.41–0.92), except for the maximum knee angle during the swing phase (ICC 0.08). ICCs for in-external rotation angles for trunk, pelvis, and hip were low to acceptable, ranging between 0 and 0.57 (Figure 3). Only for knee and ankle in/external rotation, good to excellent between-session ICCs were found (ICCknee 0.80–0.88; ICCankle 0.70–0.90). For both swing and stance phase, the between-operator ICCs of the flexion/extension angles were acceptable to excellent (ICC 0.33–0.95). For the ab/adduction angles, the ICCs of the hip, knee, and ankle angles were acceptable to good (ICC 0.36–0.87), except for the trunk angles (ICC 0.16–0.71) and pelvis angles (ICC 0.01–0.54). With regard to the in/external rotation angles, only the ICCs of the knee and ankle angle were acceptable to good (ICC 0.61–0.90) (Figure 3).

##### Forward Lunge

**Figure 4:**
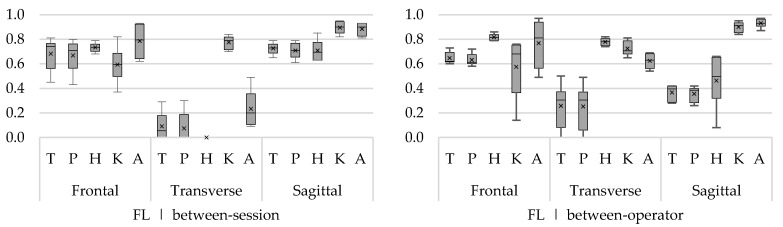
Between-session (**left**) and between-operator (**right**) reliability of minimum and maximum trunk (T), pelvis (P), hip (H), knee (K), and ankle (A) angles of the forward lunge (FL) in the frontal, transverse, and sagittal plane. The *y*-axis represents the ICC.

The between-session reliability was acceptable to good for flexion/extension angles (ICC 0.61–0.95) and the ab/adduction angles (ICC 0.43–0.93). Except for the knee ab/adduction angle during the swing backwards (SWoff), reliability was a slightly lower (ICC 0.37). Over the different phases, the between-session ICCs for in/external rotation angles of the trunk, pelvis, and hip were low (ICC 0–0.30), except for the ICCs of the knee angle, which were good (0.70–0.84) (Figure 4). Across all phases, between-operator ICCs were highest for ab/adduction angles (ICC 0.44–0.97), with the exception of the maximum knee angle during SWoff (ICC 0.14). With regard to flexion/extension angles, only ICCs for knee and ankle angles were good to excellent (ICC 0.84–0.97), while ICCs of the trunk, pelvis, and hip were substantially lower (0.08–0.66). The between-operator ICCs of the in/external rotation angles of hip, knee, and ankle were acceptable to good (ICC 0.54–0.82).

##### Sideward Lunge

**Figure 5:**
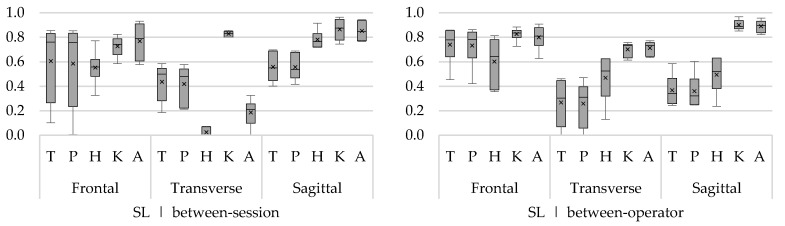
Between-session (**left**) and between-operator (**right**) reliability of minimum and maximum trunk (T), pelvis (P), hip (H), knee (K), and ankle (A) angles of the sideward lunge (SL) in the frontal, transverse, and sagittal plane. The *y*-axis represents the ICC.

Between-session reliability was acceptable to excellent for the flexion/extension angles (ICC 0.40–0.92) during all phases (Figure 5). The ab/adduction angles showed acceptable to excellent reliability (ICC 0.57–0.86) during the SWon and FTon phase, except for the maximum hip ab/adduction angle during SWon (ICC 0.33). During SWoff phase, acceptable to excellent results were found for the hip, knee, and ankle angles (ICC 0.53–0.90), while the reliability for the trunk and pelvis angles was low (0.01–0.32). Only the knee in/external rotation angle showed consistently good reliability (ICC 0.80–0.86) for all phases (Figure 5), while the reliability for the other joints was substantially lower (ICC 0.0–0.58).

The between-operator ICCs of the ab/adduction angles were acceptable to good (ICC 0.36–0.87) for the SWon and SWoff phases (Figure 5) and slightly higher for the FTon phase (ICC 0.54–0.91). Between-operator ICCs of the flexion/extension angles were good to excellent for the knee and ankle angles (ICC 0.82–0.96) and substantially lower for the trunk, pelvis, and hip angles (ICC 0.24–0.63) across all phases. Also for knee and ankle in/external rotation angles, acceptable to good between-operator ICCs were found (0.61–0.77).

##### Upstairs

**Figure 6:**
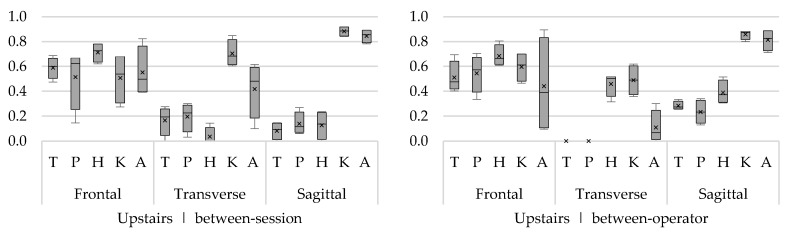
Between-session (**left**) and between-operator (**right**) reliability of minimum and maximum trunk (T), pelvis (P), hip (H), knee (K), and ankle (A) angles of upstairs in the frontal, transverse, and sagittal plane. The *y*-axis represents the ICC.

The between-session ICCs for the trunk, hip, and ankle ab/adduction angles were consistently acceptable to good during all phases (ICC 0.39–0.78). While for the pelvis and knee angles, only the minimum angles were acceptable (ICC 0.58–068) and the maximum angles showed low reliability (ICC 0.14–0.67). For the flexion/extension angles, only the knee and ankle joints showed consistently good to excellent reliability (ICC 0.78–0.92). Between-session ICCs of trunk, pelvis, and hip in/external rotation angles were low in general (Figure 6), acceptable ICCs were found for knee and ankle (ICC 0.52–0.85) during the swing phase and for the knee (ICC 0.60–0.65) during the stance phase. Regarding the between-operator reliability, acceptable to good ICCs were found for ab/adduction angles (ICC 0.33–0.89), with low ICCs for the minimum ankle angles during swing and stance (ICC < 0.20). The ICCs of the flexion/extension angels were only good for knee and ankle angles (ICC 0.71–0.89), while trunk, pelvis, and hip angles were substantially lower (ICC 0.13–0.51). The between-operator reliability of the in/external rotation angles was low in general (Figure 6), with the highest ICCs for the hip (ICC 0.32–0.52) and knee (ICC 0.36–0.62) in/external rotation angles.

##### Downstairs

**Figure 7:**
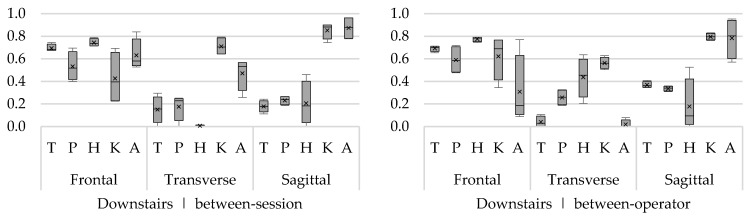
Between-session (**left**) and between-operator (**right**) reliability of minimum and maximum trunk (T), pelvis (P), hip (H), knee (K), and ankle (A) angles of downstairs in the frontal plane (F), transverse plane (T), and sagittal plane (S). The *y*-axis represents the ICC.

Between-session ICCs were highest for the ab/adduction angles (ICC 0.40–0.84), except for the maximum knee ab/adduction angles (ICC < 0.25). For flexion/extension, only for the knee and ankle angles, good to excellent results (ICC 0.74–0.96) were found across all phases. For in/external rotation angles, acceptable ICCs were found in the knee (ICC 0.64–0.79). For the other angles, the between-session ICCs were substantially lower (ICC 0.0–0.57) (Figure 7). With regard to the between-operator reliability, acceptable to good ICCs were found for ab/adduction angles (ICC 0.39–0.78) in all joints and phases, except for the ankle angles (ICC 0.09–0.77). The between-operator ICCs of flexion/extension angels were only good to excellent for the knee angles (ICC 0.79–0.95) and acceptable to good for the ankle angles (ICC 0.57–0.95). The between-operator ICCs for transverse plane angles were low (Figure 7), with acceptable ICCs only for the knee (0.50–0.63).

#### 3.2.3. Agreement

**Figure 8:**
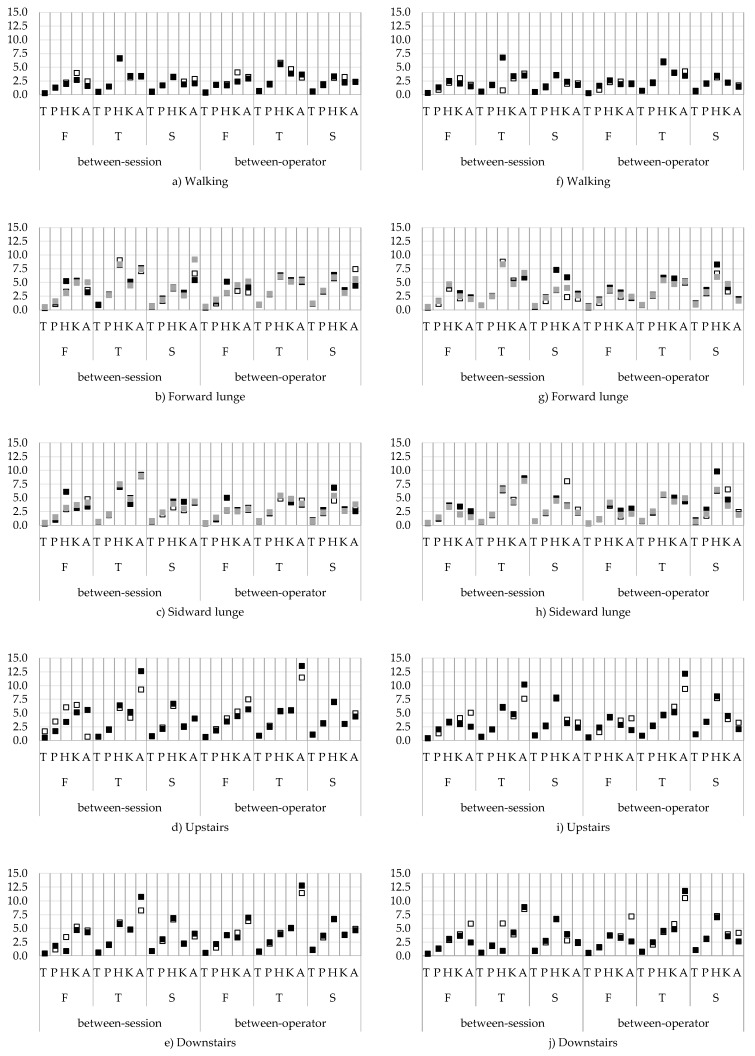
Between-session and between-operator standard errors of measurement (SEMs) for the minimum (a–e) and maximum (f–j) joint angles from all tasks for the swing/swing towards foot contact (white), stance/foot contact on the ground (black), and swing backwards (grey). On the *x*-axis the movement planes: frontal plane (F), transverse plane (T), and sagittal plane (S), and the different joints: trunk (T), pelvis (P), hip (H), knee (K), and ankle (A). The *y*-axis represents the SEM in degrees.

The between-session and between-operator SEMs are presented in Figure 8a–j. For the between-session and between-operator agreement the trunk and pelvis angles showed the highest agreement (SEM < 5.0°) for all tasks and for all planes, while the SEMs of the hip, knee, and ankle angles were larger, that is, up to 13.5° (Figure 8d). Between-session SEMs for hip, knee, and ankle ab-adduction angles were between 1.4 and 6.5° for all tasks. Of the flexion/extension angles, the hip angles showed the highest SEMs, especially during the forward and sideward lunges and upstairs walking (Figure 8b–e). For the in/external rotation angles, SEMs were larger; for walking and forward/sideward lunge, between 0.8 and 9.2°, and for up and downstairs, up to 12.6° (Figure 8e).

For the between-operator agreement (Figure 8f–j), the highest SEMs were found for the in/external rotation angles. Especially for up and downstairs, the SEMs of the in/external rotation ankle angles went up to 13.5° (Figure 8i,j). Additionally, the hip flexion/extension angles showed the highest SEM up to 9.8° during FTon phase of the forward and sideward lunge and up to 8.0° for going upstairs (Figure 8g–i). Ab/adduction angles showed the lowest SEMs (1.6–7.5°). Individual SEMs and MDC from all activities and all planes are provided in the supplementary materials.

## 4. Discussion

This study aimed to determine the reliability and agreement of the trunk and lower limb 3D kinematics, assessed by means of an inertial sensor system, during the performance of physically demanding tasks (i.e., for persons with degenerative disorders) in addition to standard gait analysis. Furthermore, reliability and agreement were determined for different phases of the movement tasks (i.e., open versus closed chain) and these results were used to provide recommendations for parameter selection in future research.

Within-session reliability and agreement results were consistently high for all tasks and phases. Therefore, the between-session and between-operator reliability and agreement were further assessed. In general, for all tasks, the between-session reliability was slightly better than the between-operator reliability, but always lower compared with the within-session reliability (Figure S11). Better between-session than between-operator results can be due to operator-dependent sources of variation, such as differences in sensor placement, differences in calibration procedure, or differences in the explanation of task execution.

### 4.1. Interpretation of Study Results

Walking showed the best results regarding the between-session and between-operator reliability and agreement, and no differences were obtained between the swing and stance phase. With the exception of the hip in-external rotation angles, walking was the only task that showed good results for the in-external rotation angles. However, as walking is a cyclic and repetitive movement, it is supposed that for the assessment of (mal)adaptive movement strategies more challenging and demanding tasks are required [10]. The present study showed that the reliability of the ab/adduction and flexion/extension angles during the forward lunge (all phases) and sideward lunge (SWon and FTon phases) were comparable to the results of walking. However, the corresponding SEMs of the hip (up to 9.8°) and ankle (up to 9.2°) flexion/extension angles were higher when compared with the reported SEMs during walking (SEM < 4°). This could potentially be explained by the fact that in the performance of the forward and sideward lunge, a larger hip and ankle motion is required when compared with walking. Based on these results and the lower within-session ICCs for the SWoff phase, it would be recommended to use the SWon phase to assess open-chain kinematics, instead of the SWoff phase. The lower reliability of SWoff is possibly caused by the fact that this movement requires more motor control and force to extent the knee to perform the backwards moving, which is in contrast to the SWon, which starts with a forward swing of the leg until the foot makes contact with the ground. Furthermore, the reliability and agreement of the in/external rotation angle was much lower across joints and phases. This could be explained by the fact that only the step length was standardized, allowing for a variable performance of the lunge in terms of hip, knee, and ankle rotations in the transversal plane. Greater variation in the in/external rotational angles of hip, knee, and ankle joints was also observed in the waveforms (Supplementary Materials Figures S6–S10).

Compared with walking and both lunges, going up and downstairs showed the lowest reliability. Especially for the in/external rotation (ICC 0–0.64) and flexion/extension (ICC 0–0.51) trunk, pelvis, and hip angles, the reliability was low. Moreover, the error of the flexion/extension (SEM up to 8.0°) and in/external rotation angles (SEM up to 13.5°) were high. Based on these results it is not recommended to assess (mal)adaptive movement strategies while going up and downstairs, as these are not reliable and show the greatest errors, especially when it is know that the degree of reliability should be determined in the context of the specific use. Errors greater than five degrees could lead to misinterpretation of the results and require explicit data interpretation [19,20]. For both lunges, the highest errors (SEM up to 9°) were found in the hip and ankle flexion/extension angles. However, these rotations also have a large range of motion. In contrast, for the in/external rotational angles during up and downstairs tasks, high errors for a small range of motion were obtained. Because the errors for up and downstairs are not proportional to the range of motion, these tasks are not recommended to be included for the assessment of (mal)adaptive movement strategies.

Previous studies regarding the reliability of kinematics by means of an inertial sensor system investigated the (functional) calibration procedure [13,21,22,23] or the assessment of lower limb joint kinematics in one or multiple movement planes [8,9]. Good reliability with small errors (<5°) were reported for flexion/extension angles and ab/adduction angles [8,9,13,21,22,23], in contrast to the in/external rotation angles, which showed larger errors, especially for the ankle angle (5–20°) [8,22,23]. Although these results were obtained during level walking and with respect to the reported error, the results of the present study are in line with these findings. Moreover, no differences were found between the swing and stance phase during walking in the present study. The study of Neüsh et al. reported lower ICCs in the first half of the stride (i.e., stance phase) compared with the second half of the stride (i.e., swing phase), which indicates potential differences between the open and closed chain kinematics during walking [9]. However, the results of the present study give no indication on differences in ICC or SEM between the stance and swing phase kinematics. Furthermore, the study of Al-amri et al. examined more complex and dynamic tasks [7]. In addition, they showed that the reliability of walking and squatting was acceptable, but for more dynamic tasks, such as a vertical jump, the reliability dropped and errors increased. Further standardization of movements was recommended by these authors [7]. However, caution is required as the standardization may not interfere with natural movement behaviour of the participant. Besides the instruction of the task execution, it is thus recommended to standardize the sensor positioning and calibration procedure in order to further improve the reliability and agreement of challenging and demanding tasks.

### 4.2. Study Limitations and Future Research

Within the present study, the between-session and between-operator reliability and agreement were lower compared with the within-session results. Despite the fact that everything had been done to reduce the operator-dependent sources of variation and standardize the task execution, differences between sessions and operators occurred. The calibration procedure and calibration position is essential in order to measure accurate and reliable joint angles [13]. Robert-Lachaine et al. showed that it is important to position the participant passively in the correct position in order to reduce offset in the data [13]. For this study, an N-pose calibration was performed to align the sensors to the segments [3]. Participants were passively positioned in the correct position and instructed to stand still for several seconds. This is no problem for healthy participants who do not suffer from range of motion deficits. However, in future studies including persons with pathology, it will potentially not be able to position the patient in the proposed position and to hold this for several seconds. Therefore, further research is required to improve the calibration procedure, for example, by including a seated or dynamic calibration.

Despite that participants were positioned passively in the correct position, small deviations might have been occurred in the position of the pelvis sensor (L5/S1), which is essential for a reliable measurement of the hip and trunk kinematics. Therefore, when positioning the participant in the N-pose, it should be checked if the pelvis position is in a neutral position. Furthermore, it is recommended to check the position of the pelvis sensor in between dynamical tasks, as it was noticed that the position of the sensor is easily altered by the shorts that moves against the sensor. For longitudinal data collection, it is thus recommended that data is collected by one operator (i.e., the same person). Future research should additionally investigate if lower reliability for the between-session and operator occurs as a result of variation in task execution (i.e., natural movement variability) or is induced by inconsistency of the inertial sensor system itself. Therefore, the trunk and lower limb joints’ 3D kinematics of an inertial sensor system during complex and dynamical task performance should be compared against an optoelectronic system (i.e., gold standard), before it can be used to assess (mal)adaptive movement strategies in persons with pathology (e.g., degenerative knee disorders).

## 5. Conclusions

For all tasks, phases, and rotations, high within-session reliability and agreement were found. Between-session results were generally better compared with the between-operator results; however, both were substantially lower compared with the within-session reliability and agreement. In the case that the evaluation of 3D trunk and lower limb joint kinematics is of interest, the best option is to perform a walking task, as walking showed acceptable to excellent results for all phases and rotations. When more physically demanding tasks are required to identify (mal)adaptive movement, the forward lunge and sideward lunges could be used in further research to identify kinematic differences between populations. In addition, further research should focus on the comparison of joint kinematics measured by means of an internal sensor system with an optoelectronic system (i.e., gold standard).

## Figures and Tables

**Table 1:** Detailed description of the instructions to the participants.

Task	Instruction
Walking	Start in an upright position, with the feet aligned to the marked starting line. Walk at comfortable speed as you would normally do to the other side of the lab (10 m) until you have passed the stopping line.
Forward lunge	Start in an upright position, keep your hands slightly away from the body and the toes aligned with the marked starting line. Step forward with your heel over the predetermined distance (70% leg length) as marked on the ground. While stepping forward, bring your weight of the upper body over the leading leg and be sure that the contralateral leg keeps in contact with the floor throughout the forward lunge. Make sure the entire foot contacts the ground and subsequently step backwards to the initial start position.
Sideward lunge	Start in an upright position, keep your hands slightly away from the body and the side of the foot aligned with the marked starting line. Step sideward with your foot over the predetermined distance (70% leg length) as marked on the ground and keep the foot parallel to the marked line. While stepping sideward, bring your weight of the upper body over the leading leg and be sure that the contralateral leg keeps in contact with the floor throughout the sideward lunge. Make sure the entire foot makes contact with the ground and subsequently step backwards to the initial start position.
Upstairs/Downstairs	Start in an upright position, with the feet aligned next to each other in front of the first step. Ascent the stair and wait on top of the staircase until we have given the instruction to turn around. At our sign, descent the stair and wait at the bottom of the stair until you’re instructed to turn around.

**Table 2:** Description of functional movements with division of sub-phases.

Movement	Sub-Phase	Definition Sub-Phases
Walking	Stance (ST) Swing (SW)	Initial contact until toe-off Toe-off until initial foot contact
Forward lunge/sideward lunge	Swing towards foot contact (SWon) Foot contact on the ground (FTon) Swing backwards (SWoff)	Toe-off until initial foot contact Foot contact until contact was terminated Foot off until foot contact at initial start
Upstairs	Stance (ST) Swing (SW)	2nd stair step up initial contact until toe-off 2nd stair step up toe off until 4th step initial contact
Downstairs	Stance (ST) Swing (SW)	2nd stair step down initial contact until toe-off 2nd stair step down toe off until 4th step initial contact

## Supplementary Materials

The following are available online at http://www.mdpi.com/1424-8220/18/8/2638/s1. The individual results from the ICCs, SEMs, and MDCs from all tasks are provided in Tables S1–S5. Waveforms for each task, phase and rotation are provided in Figures S6–S10. A visual comparison of the within-session, between-session, and between-operator ICCs per rotation for all tasks is provided in Figure S11.
