# Supplementary material for: Reliability of 3D Lower Extremity Movement Analysis by Means of Inertial Sensor Technology during Transitional Tasks
Source: Sensors (Basel). 2018 Aug 11;18(8):2638. doi: 10.3390/s18082638 (PMC6111309; doi:10.3390/s18082638)
Supplement: Supplementary file 1 [file sensors-18-02638-s001.pdf]

Table S1: Between-session and between-operator reliability (ICC, confidence intervals) and agreement (SEM, MDC) of joint angles from walking.

			minSW				minST				maxSW				maxST			
			ICC	CI	SEM	MDC	ICC	CI	SEM	MDC	ICC	CI	SEM	MDC	ICC	CI	SEM	MDC
within-session	frontal	Trunk	0,91	(0,84 - 0,96)	0,1	0,4	0,88	(0,78 - 0,95)	0,1	0,4	0,89	(0,8 - 0,95)	0,1	0,4	0,87	(0,75 - 0,94)	0,1	0,4
		Pelvis	0,90	(0,81 - 0,96)	0,7	2,0	0,92	(0,84 - 0,96)	0,6	1,6	0,79	(0,63 - 0,9)	0,5	1,4	0,78	(0,62 - 0,9)	0,8	2,3
		Hip	0,86	(0,74 - 0,94)	1,1	3,0	0,77	(0,6 - 0,9)	1,0	2,8	0,94	(0,87 - 0,97)	0,7	1,9	0,89	(0,8 - 0,95)	1,1	3,1
		Knee	0,97	(0,94 - 0,99)	1,1	3,0	0,95	(0,9 - 0,98)	0,8	2,3	0,96	(0,93 - 0,98)	0,7	2,0	0,94	(0,88 - 0,97)	0,7	1,9
		Ankle	0,81	(0,66 - 0,91)	1,9	5,3	0,80	(0,64 - 0,91)	2,0	5,5	0,97	(0,94 - 0,99)	0,9	2,5	0,88	(0,77 - 0,95)	1,3	3,6
	transverse	Trunk	0,91	(0,82 - 0,96)	0,2	0,6	0,93	(0,86 - 0,97)	0,2	0,5	0,90	(0,8 - 0,96)	0,2	0,6	0,91	(0,82 - 0,96)	0,2	0,6
		Pelvis	0,90	(0,81 - 0,96)	0,6	1,8	0,92	(0,84 - 0,96)	0,6	1,8	0,88	(0,77 - 0,95)	0,8	2,1	0,90	(0,81 - 0,96)	0,7	1,9
		Hip	0,96	(0,92 - 0,98)	1,5	4,2	0,95	(0,91 - 0,98)	1,6	4,5	0,95	(0,91 - 0,98)	1,6	4,4	0,92	(0,85 - 0,97)	1,7	4,7
		Knee	0,95	(0,9 - 0,98)	1,7	4,7	0,96	(0,91 - 0,98)	1,5	4,1	0,95	(0,9 - 0,98)	1,4	4,0	0,94	(0,88 - 0,97)	1,6	4,4
		Ankle	0,94	(0,86 - 0,98)	1,6	4,4	0,91	(0,82 - 0,96)	1,7	4,7	0,93	(0,86 - 0,97)	2,7	7,5	0,86	(0,74 - 0,94)	2,9	7,9
	sagittal	Trunk	0,93	(0,87 - 0,97)	0,2	0,6	0,91	(0,83 - 0,96)	0,2	0,6	0,94	(0,88 - 0,98)	0,2	0,5	0,94	(0,89 - 0,98)	0,2	0,6
		Pelvis	0,93	(0,87 - 0,97)	0,7	1,8	0,90	(0,82 - 0,96)	0,7	1,9	0,94	(0,87 - 0,97)	0,6	1,8	0,92	(0,85 - 0,97)	0,6	1,7
		Hip	0,92	(0,85 - 0,97)	1,4	3,8	0,93	(0,85 - 0,97)	1,2	3,4	0,92	(0,85 - 0,97)	1,5	4,0	0,90	(0,81 - 0,96)	2,1	5,8
		Knee	0,92	(0,85 - 0,97)	1,7	4,8	0,90	(0,81 - 0,96)	1,7	4,7	0,94	(0,87 - 0,97)	1,5	4,1	0,86	(0,74 - 0,94)	2,3	6,4
		Ankle	0,93	(0,86 - 0,97)	2,1	5,9	0,81	(0,66 - 0,91)	1,9	5,4	0,88	(0,77 - 0,95)	1,8	5,0	0,91	(0,83 - 0,96)	1,2	3,3
between-session	frontal	Trunk	0,69	(0,19 - 0,88)	0,3	0,7	0,59	(0 - 0,84)	0,3	0,8	0,65	(0,12 - 0,86)	0,3	0,8	0,65	(0,1 - 0,86)	0,3	0,8
		Pelvis	0,68	(0,17 - 0,87)	1,2	3,4	0,48	(0 - 0,8)	1,3	3,6	0,68	(0,19 - 0,87)	0,9	2,5	0,64	(0,1 - 0,85)	1,3	3,6
		Hip	0,55	(0 - 0,82)	2,2	6,1	0,42	(0 - 0,77)	1,9	5,4	0,62	(0,01 - 0,85)	2,1	6,0	0,49	(0 - 0,8)	2,5	7,0
		Knee	0,65	(0,13 - 0,86)	3,9	10,9	0,71	(0,27 - 0,88)	2,7	7,4	0,08	(0 - 0,65)	3,0	8,3	0,41	(0 - 0,76)	2,0	5,6
		Ankle	0,76	(0,39 - 0,91)	2,4	6,7	0,92	(0,79 - 0,97)	1,6	4,3	0,91	(0,79 - 0,97)	1,8	5,0	0,85	(0,62 - 0,94)	1,5	4,2
	transverse	Trunk	0,45	(0 - 0,79)	0,5	1,4	0,49	(0 - 0,8)	0,5	1,4	0,54	(0 - 0,82)	0,6	1,6	0,57	(0 - 0,83)	0,6	1,6
		Pelvis	0,48	(0 - 0,8)	1,5	4,1	0,50	(0 - 0,8)	1,5	4,1	0,50	(0 - 0,8)	1,8	5,0	0,57	(0 - 0,83)	1,7	4,8
		Hip	0,02	(0 - 0,62)	6,6	18,4	0,00	(0 - 0,6)	6,6	18,2	0,00	(0 - 0,39)	0,8	2,2	0,00	(0 - 0,39)	6,7	18,7
		Knee	0,88	(0,67 - 0,95)	3,2	8,7	0,81	(0,53 - 0,92)	3,4	9,4	0,85	(0,63 - 0,94)	3,0	8,3	0,80	(0,5 - 0,92)	3,4	9,4
		Ankle	0,79	(0,46 - 0,92)	3,3	9,2	0,70	(0,27 - 0,88)	3,4	9,4	0,90	(0,75 - 0,96)	3,8	10,6	0,85	(0,63 - 0,94)	3,5	9,6
	sagittal	Trunk	0,75	(0,36 - 0,9)	0,5	1,4	0,63	(0,05 - 0,86)	0,6	1,5	0,82	(0,53 - 0,93)	0,5	1,4	0,81	(0,5 - 0,92)	0,5	1,4
		Pelvis	0,70	(0,22 - 0,88)	1,7	4,7	0,62	(0,02 - 0,85)	1,7	4,7	0,82	(0,53 - 0,93)	1,5	4,2	0,80	(0,5 - 0,92)	1,3	3,7
		Hip	0,63	(0,1 - 0,85)	3,3	9,0	0,57	(0 - 0,83)	3,2	8,8	0,60	(0,02 - 0,84)	3,6	9,9	0,77	(0,42 - 0,91)	3,5	9,7
		Knee	0,90	(0,73 - 0,96)	2,4	6,6	0,93	(0,83 - 0,97)	1,9	5,2	0,88	(0,48 - 0,96)	2,0	5,5	0,91	(0,76 - 0,96)	2,4	6,5
		Ankle	0,94	(0,84 - 0,97)	2,9	8,0	0,90	(0,74 - 0,96)	2,1	5,7	0,92	(0,8 - 0,97)	2,0	5,7	0,90	(0,76 - 0,93)	1,8	4,9
between-operator	frontal	Trunk	0,34	(0 - 0,76)	0,4	1,0	0,16	(0 - 0,69)	0,4	1,2	0,67	(0,12 - 0,88)	0,3	0,7	0,71	(0 - 0,89)	0,3	0,7
		Pelvis	0,12	(0 - 0,68)	1,7	4,8	0,01	(0 - 0,64)	1,8	5,0	0,54	(0 - 0,83)	0,9	2,5	0,25	(0 - 0,73)	1,6	4,4
		Hip	0,73	(0,27 - 0,9)	1,9	5,3	0,44	(0 - 0,79)	1,7	4,7	0,39	(0 - 0,77)	2,3	6,3	0,53	(0 - 0,83)	2,6	7,2
		Knee	0,70	(0,2 - 0,89)	4,1	11,3	0,84	(0,56 - 0,94)	2,4	6,6	0,55	(0 - 0,83)	2,4	6,6	0,36	(0 - 0,77)	1,9	5,3
		Ankle	0,49	(0 - 0,8)	3,2	8,9	0,65	(0,1 - 0,87)	2,9	8,2	0,87	(0,64 - 0,95)	2,1	5,7	0,80	(0,45 - 0,93)	1,9	5,2
	transverse	Trunk	0,49	(0 - 0,81)	0,6	1,7	0,40	(0 - 0,77)	0,7	1,9	0,44	(0 - 0,8)	0,7	2,0	0,48	(0 - 0,81)	0,7	2,0
		Pelvis	0,45	(0 - 0,79)	1,8	5,1	0,40	(0 - 0,77)	2,0	5,5	0,43	(0 - 0,79)	2,2	6,2	0,49	(0 - 0,81)	2,1	5,8
		Hip	0,27	(0 - 0,73)	5,8	16,1	0,30	(0 - 0,74)	5,6	15,4	0,00	(0 - 0,57)	6,0	16,8	0,00	(0 - 0,18)	5,9	16,3
		Knee	0,66	(0,14 - 0,87)	4,6	12,8	0,66	(0,14 - 0,87)	3,8	10,7	0,65	(0,12 - 0,87)	4,0	11,0	0,61	(0,04 - 0,85)	4,0	11,2
		Ankle	0,79	(0,44 - 0,92)	3,1	8,6	0,62	(0 - 0,86)	3,7	10,1	0,90	(0,73 - 0,96)	4,2	11,7	0,86	(0,62 - 0,95)	3,5	9,6
	sagittal	Trunk	0,60	(0 - 0,85)	0,6	1,6	0,42	(0 - 0,79)	0,6	1,7	0,62	(0 - 0,86)	0,6	1,7	0,52	(0 - 0,82)	0,7	2,0
		Pelvis	0,58	(0 - 0,84)	1,7	4,8	0,33	(0 - 0,76)	1,9	5,3	0,52	(0 - 0,82)	2,0	5,5	0,44	(0 - 0,79)	2,0	5,7
		Hip	0,67	(0,12 - 0,87)	3,1	8,5	0,43	(0 - 0,79)	3,3	9,2	0,79	(0,47 - 0,92)	3,1	8,7	0,84	(0,59 - 0,94)	3,4	9,6
		Knee	0,84	(0,58 - 0,94)	3,2	8,9	0,89	(0,71 - 0,96)	2,2	6,1	0,90	(0,7 - 0,96)	2,2	6,1	0,93	(0,82 - 0,98)	2,2	6,0
		Ankle	0,95	(0,86 - 0,98)	2,3	6,5	0,81	(0,49 - 0,93)	2,3	6,4	0,93	(0,82 - 0,97)	1,7	4,7	0,93	(0,81 - 0,97)	1,4	3,9

Table S2: Between-session and between-operator reliability (ICC, confidence intervals) and agreement (SEM, MDC) of joint angles from forward lunge.

			minSWon				minFTon				minSWoff				maxSWon				maxFTon				maxSWoff			
			ICC	CI	SEM	MDC	ICC	CI	SEM	MDC	ICC	CI	SEM	MDC	ICC	CI	SEM	MDC	ICC	CI	SEM	MDC	ICC	CI	SEM	MDC
within-session	frontal	Trunk	0,89	(0,78 - 0,95)	0,2	0,6	0,81	(0,65 - 0,92)	0,3	1,0	0,77	(0,59 - 0,9)	0,5	1,4	0,94	(0,88 - 0,98)	0,1	0,4	0,83	(0,69 - 0,93)	0,3	0,9	0,85	(0,71 - 0,94)	0,4	1,2
		Pelvis	0,88	(0,77 - 0,95)	0,6	1,7	0,78	(0,6 - 0,91)	1,2	3,3	0,79	(0,61 - 0,91)	1,5	4,1	0,93	(0,86 - 0,97)	0,4	1,2	0,85	(0,7 - 0,94)	1,0	2,9	0,86	(0,72 - 0,94)	1,2	3,4
		Hip	0,96	(0,92 - 0,98)	1,1	3,0	0,94	(0,87 - 0,98)	1,8	5,0	0,90	(0,79 - 0,96)	2,0	5,7	0,96	(0,91 - 0,98)	1,0	2,9	0,92	(0,84 - 0,97)	1,4	4,0	0,86	(0,73 - 0,94)	2,0	5,5
		Knee	0,96	(0,92 - 0,99)	1,0	2,8	0,94	(0,88 - 0,98)	1,4	3,9	0,92	(0,84 - 0,97)	1,6	4,6	0,97	(0,93 - 0,99)	0,6	1,8	0,88	(0,77 - 0,95)	1,4	3,9	0,86	(0,74 - 0,95)	1,1	3,0
		Ankle	0,88	(0,75 - 0,95)	2,0	5,5	0,83	(0,67 - 0,93)	2,1	5,9	0,83	(0,68 - 0,93)	2,9	8,0	0,93	(0,85 - 0,97)	1,7	4,6	0,83	(0,69 - 0,93)	2,7	7,5	0,91	(0,81 - 0,96)	1,7	4,7
	transverse	Trunk	0,98	(0,96 - 0,99)	0,2	0,5	0,98	(0,95 - 0,99)	0,2	0,6	0,94	(0,87 - 0,97)	0,3	0,8	0,98	(0,96 - 0,99)	0,1	0,4	0,94	(0,87 - 0,98)	0,3	0,8	0,93	(0,85 - 0,97)	0,3	0,9
		Pelvis	0,98	(0,97 - 0,99)	0,5	1,4	0,97	(0,94 - 0,99)	0,6	1,8	0,92	(0,84 - 0,97)	1,0	2,7	0,98	(0,96 - 0,99)	0,4	1,2	0,94	(0,87 - 0,98)	0,8	2,3	0,92	(0,85 - 0,97)	0,9	2,6
		Hip	0,98	(0,96 - 0,99)	1,4	4,0	0,97	(0,94 - 0,99)	1,4	4,0	0,96	(0,91 - 0,98)	2,0	5,5	0,97	(0,93 - 0,99)	1,6	4,5	0,98	(0,96 - 0,99)	1,4	3,9	0,98	(0,95 - 0,99)	1,6	4,5
		Knee	0,94	(0,87 - 0,98)	2,2	6,2	0,93	(0,85 - 0,97)	2,5	7,0	0,91	(0,81 - 0,96)	2,4	6,6	0,97	(0,94 - 0,99)	1,6	4,6	0,95	(0,91 - 0,98)	2,1	5,9	0,97	(0,93 - 0,99)	1,7	4,7
		Ankle	0,96	(0,92 - 0,99)	1,4	3,8	0,93	(0,85 - 0,97)	2,0	5,6	0,93	(0,86 - 0,97)	1,8	5,0	0,93	(0,86 - 0,97)	1,9	5,3	0,82	(0,67 - 0,93)	2,7	7,5	0,80	(0,63 - 0,92)	3,2	8,8
	sagittal	Trunk	0,97	(0,93 - 0,99)	0,2	0,5	0,96	(0,92 - 0,98)	0,2	0,6	0,97	(0,93 - 0,99)	0,2	0,6	0,96	(0,92 - 0,99)	0,2	0,5	0,96	(0,91 - 0,98)	0,2	0,6	0,95	(0,9 - 0,99)	0,2	0,6
		Pelvis	0,97	(0,93 - 0,99)	0,6	1,6	0,96	(0,91 - 0,98)	0,6	1,8	0,96	(0,91 - 0,98)	0,7	1,9	0,96	(0,92 - 0,99)	0,5	1,5	0,96	(0,93 - 0,99)	0,6	1,8	0,95	(0,89 - 0,98)	0,8	2,1
		Hip	0,81	(0,64 - 0,92)	1,9	5,4	0,91	(0,82 - 0,96)	1,9	5,2	0,63	(0,4 - 0,83)	3,9	10,9	0,91	(0,82 - 0,97)	2,3	6,4	0,95	(0,89 - 0,98)	1,9	5,2	0,89	(0,78 - 0,96)	1,9	5,4
		Knee	0,95	(0,9 - 0,98)	1,5	4,1	0,79	(0,61 - 0,91)	3,1	8,6	0,84	(0,7 - 0,94)	2,5	7,0	0,90	(0,81 - 0,96)	3,3	9,0	0,93	(0,85 - 0,97)	3,6	10,0	0,89	(0,77 - 0,95)	4,6	12,9
		Ankle	0,81	(0,64 - 0,92)	7,1	19,6	0,72	(0,51 - 0,88)	5,6	15,4	0,77	(0,58 - 0,9)	7,9	21,9	0,89	(0,79 - 0,96)	1,8	5,0	0,94	(0,88 - 0,98)	2,1	5,8	0,95	(0,91 - 0,98)	1,4	3,9
between-session	frontal	Trunk	0,60	(0 - 0,84)	0,3	0,9	0,75	(0,38 - 0,9)	0,4	1,1	0,81	(0,53 - 0,92)	0,5	1,4	0,45	(0 - 0,78)	0,4	1,0	0,75	(0,36 - 0,9)	0,5	1,5	0,73	(0,34 - 0,89)	0,5	1,5
		Pelvis	0,61	(0 - 0,85)	1,0	2,9	0,73	(0,33 - 0,89)	1,3	3,7	0,80	(0,5 - 0,92)	1,5	4,3	0,43	(0 - 0,78)	1,1	3,0	0,69	(0,2 - 0,88)	1,6	4,5	0,75	(0,38 - 0,9)	1,6	4,4
		Hip	0,72	(0,3 - 0,89)	3,3	9,2	0,75	(0,38 - 0,9)	5,2	14,5	0,79	(0,46 - 0,92)	3,0	8,5	0,75	(0,37 - 0,9)	3,9	10,7	0,71	(0,25 - 0,88)	4,4	12,3	0,68	(0,19 - 0,87)	4,6	12,9
		Knee	0,54	(0 - 0,82)	5,0	13,8	0,64	(0,1 - 0,86)	5,3	14,7	0,61	(0,02 - 0,85)	5,0	13,9	0,82	(0,56 - 0,93)	2,1	5,8	0,58	(0 - 0,83)	3,0	8,5	0,37	(0 - 0,75)	2,5	6,8
		Ankle	0,62	(0,09 - 0,85)	3,6	10,1	0,71	(0,27 - 0,88)	3,2	8,9	0,65	(0,09 - 0,86)	5,0	13,9	0,92	(0,75 - 0,97)	2,0	5,5	0,93	(0,82 - 0,97)	2,3	6,3	0,88	(0,65 - 0,96)	1,9	5,4
	transverse	Trunk	0,29	(0 - 0,72)	0,9	2,6	0,11	(0 - 0,64)	0,9	2,4	0,00	(0 - 0,49)	0,9	2,5	0,00	(0 - 0,49)	0,8	2,3	0,14	(0 - 0,65)	0,8	2,3	0,00	(0 - 0,58)	0,8	2,3
		Pelvis	0,30	(0 - 0,72)	2,8	7,7	0,00	(0 - 0,59)	2,7	7,4	0,00	(0 - 0,44)	2,7	7,4	0,00	(0 - 0,48)	2,5	7,1	0,15	(0 - 0,65)	2,5	6,9	0,00	(0 - 0,58)	2,5	6,8
		Hip	0,00	(0 - 0,51)	9,0	25,0	0,00	(0 - 0,31)	8,2	22,7	0,00	(0 - 0,44)	8,3	22,9	0,00	(0 - 0,21)	8,8	24,3	0,00	(0 - 0,43)	8,2	22,7	0,00	(0 - 0,5)	8,3	22,9
		Knee	0,72	(0,29 - 0,89)	5,1	14,2	0,80	(0,5 - 0,92)	5,0	13,9	0,78	(0,43 - 0,91)	4,4	12,2	0,70	(0,23 - 0,88)	5,3	14,8	0,84	(0,6 - 0,94)	5,0	13,8	0,81	(0,51 - 0,92)	4,7	12,9
		Ankle	0,12	(0 - 0,64)	7,1	19,6	0,11	(0 - 0,59)	7,6	21,1	0,28	(0 - 0,71)	7,4	20,5	0,09	(0 - 0,59)	6,5	18,0	0,31	(0 - 0,71)	5,9	16,3	0,49	(0 - 0,79)	6,7	18,7
	sagittal	Trunk	0,75	(0,38 - 0,9)	0,5	1,5	0,70	(0,23 - 0,88)	0,7	1,8	0,75	(0,37 - 0,9)	0,6	1,7	0,79	(0,48 - 0,92)	0,5	1,5	0,71	(0,27 - 0,88)	0,7	2,0	0,65	(0,11 - 0,86)	0,7	2,0
		Pelvis	0,76	(0,39 - 0,9)	1,6	4,6	0,67	(0,16 - 0,87)	2,0	5,7	0,75	(0,36 - 0,9)	1,8	5,1	0,79	(0,48 - 0,92)	1,6	4,4	0,67	(0,19 - 0,87)	2,3	6,4	0,61	(0 - 0,85)	2,2	6,1
		Hip	0,63	(0,09 - 0,85)	3,9	10,8	0,63	(0,09 - 0,85)	4,1	11,3	0,71	(0,29 - 0,88)	4,0	11,1	0,85	(0,64 - 0,94)	3,6	9,9	0,68	(0,2 - 0,87)	7,3	20,2	0,75	(0,38 - 0,9)	3,7	10,2
		Knee	0,90	(0,74 - 0,96)	2,7	7,5	0,82	(0,5 - 0,93)	3,1	8,7	0,86	(0,65 - 0,95)	2,6	7,3	0,95	(0,77 - 0,98)	2,3	6,4	0,90	(0,75 - 0,96)	5,9	16,4	0,94	(0,83 - 0,98)	4,0	11,1
		Ankle	0,87	(0,61 - 0,95)	6,6	18,4	0,83	(0,57 - 0,93)	5,4	15,0	0,81	(0,51 - 0,92)	9,2	25,4	0,93	(0,83 - 0,97)	2,0	5,6	0,93	(0,82 - 0,97)	2,9	8,2	0,93	(0,82 - 0,97)	2,6	7,3
between-operator	frontal	Trunk	0,68	(0,21 - 0,87)	0,4	1,0	0,60	(0 - 0,84)	0,5	1,4	0,62	(0,09 - 0,85)	0,6	1,6	0,62	(0,02 - 0,85)	0,4	1,1	0,62	(0,02 - 0,85)	0,7	1,8	0,73	(0,31 - 0,89)	0,5	1,5
		Pelvis	0,66	(0,14 - 0,87)	1,2	3,2	0,58	(0 - 0,83)	1,5	4,2	0,61	(0,04 - 0,84)	1,8	5,1	0,61	(0 - 0,85)	1,3	3,5	0,61	(0 - 0,85)	1,9	5,3	0,72	(0,29 - 0,89)	1,7	4,7
		Hip	0,82	(0,53 - 0,93)	3,0	8,5	0,79	(0,46 - 0,91)	5,1	14,2	0,81	(0,53 - 0,93)	3,1	8,5	0,86	(0,63 - 0,94)	3,5	9,8	0,79	(0,47 - 0,92)	4,0	11,2	0,83	(0,58 - 0,93)	3,6	10,1
		Knee	0,75	(0,36 - 0,9)	3,4	9,5	0,76	(0,41 - 0,9)	4,3	11,9	0,66	(0,13 - 0,87)	4,5	12,5	0,70	(0,22 - 0,88)	2,4	6,7	0,44	(0 - 0,78)	3,1	8,7	0,14	(0 - 0,67)	2,8	7,7
		Ankle	0,77	(0,44 - 0,91)	3,1	8,7	0,59	(0 - 0,84)	4,1	11,3	0,49	(0 - 0,79)	5,2	14,3	0,97	(0,81 - 0,97)	2,1	5,9	0,93	(0,83 - 0,97)	2,3	6,3	0,85	(0,63 - 0,94)	2,4	6,6
	transverse	Trunk	0,31	(0 - 0,73)	0,9	2,6	0,33	(0 - 0,74)	1,0	2,7	0,11	(0 - 0,66)	1,0	2,7	0,00	(0 - 0,57)	0,9	2,5	0,50	(0 - 0,8)	0,8	2,4	0,30	(0 - 0,73)	0,9	2,5
		Pelvis	0,32	(0 - 0,73)	2,8	7,9	0,33	(0 - 0,74)	2,9	8,0	0,08	(0 - 0,64)	2,9	8,0	0,00	(0 - 0,57)	2,8	7,6	0,49	(0 - 0,8)	2,6	7,1	0,29	(0 - 0,72)	2,7	7,4
		Hip	0,82	(0,53 - 0,93)	6,0	16,6	0,74	(0,33 - 0,9)	6,3	17,4	0,78	(0,44 - 0,91)	6,0	16,7	0,75	(0,36 - 0,9)	5,4	15,1	0,78	(0,44 - 0,91)	5,8	16,2	0,80	(0,49 - 0,92)	5,4	14,9
		Knee	0,69	(0,23 - 0,88)	5,4	15,0	0,71	(0,29 - 0,88)	5,3	14,7	0,65	(0,17 - 0,86)	5,1	14,2	0,70	(0,25 - 0,88)	5,3	14,6	0,78	(0,45 - 0,91)	5,7	15,8	0,81	(0,53 - 0,92)	4,7	13,0
		Ankle	0,57	(0 - 0,83)	5,5	15,2	0,69	(0,25 - 0,88)	5,1	14,0	0,61	(0,01 - 0,85)	5,3	14,7	0,65	(0,12 - 0,86)	4,9	13,7	0,54	(0 - 0,82)	5,2	14,5	0,68	(0,18 - 0,88)	5,2	14,3
	sagittal	Trunk	0,29	(0 - 0,72)	1,1	3,0	0,38	(0 - 0,75)	1,1	3,2	0,41	(0 - 0,76)	1,1	3,2	0,42	(0 - 0,77)	1,0	2,8	0,28	(0 - 0,7)	1,2	3,3	0,42	(0 - 0,76)	1,0	2,9
		Pelvis	0,29	(0 - 0,72)	3,3	9,2	0,39	(0 - 0,75)	3,4	9,6	0,39	(0 - 0,75)	3,5	9,6	0,42	(0 - 0,76)	3,0	8,3	0,26	(0 - 0,69)	3,6	10,0	0,38	(0 - 0,75)	3,2	8,9
		Hip	0,48	(0 - 0,79)	5,7	15,9	0,08	(0 - 0,63)	6,4	17,6	0,66	(0,16 - 0,86)	5,9	16,4	0,40	(0 - 0,76)	6,6	18,3	0,65	(0,13 - 0,86)	8,3	22,9	0,51	(0 - 0,8)	6,0	16,6
		Knee	0,90	(0,75 - 0,96)	3,2	8,9	0,84	(0,61 - 0,94)	3,6	10,0	0,86	(0,64 - 0,94)	3,1	8,5	0,93	(0,83 - 0,97)	3,3	9,3	0,95	(0,88 - 0,98)	4,1	11,4	0,92	(0,79 - 0,97)	4,7	13,1
		Ankle	0,87	(0,67 - 0,95)	7,4	20,5	0,92	(0,79 - 0,97)	4,4	12,2	0,93	(0,79 - 0,97)	5,6	15,6	0,93	(0,82 - 0,97)	1,9	5,4	0,96	(0,86 - 0,99)	1,9	5,2	0,97	(0,93 - 0,99)	1,7	4,6

Table S3: Between-session and between-operator reliability (ICC, confidence intervals) and agreement (SEM, MDC) of joint angles from sideward lunge.

			minSWon				minFTon				minSWoff				maxSWon				maxFTon				maxSWoff			
			ICC	CI	SEM	MDC	ICC	CI	SEM	MDC	ICC	CI	SEM	MDC	ICC	CI	SEM	MDC	ICC	CI	SEM	MDC	ICC	CI	SEM	MDC
within-session	frontal	Trunk	0,90	(0,82 - 0,96)	0,2	0,6	0,88	(0,78 - 0,95)	0,2	0,7	0,63	(0,42 - 0,81)	0,3	0,9	0,91	(0,84 - 0,96)	0,2	0,5	0,87	(0,76 - 0,94)	0,3	0,7	0,83	(0,7 - 0,92)	0,3	0,7
		Pelvis	0,91	(0,83 - 0,96)	0,6	1,6	0,88	(0,78 - 0,95)	0,7	2,0	0,67	(0,47 - 0,84)	0,9	2,6	0,91	(0,83 - 0,96)	0,6	1,7	0,86	(0,74 - 0,93)	0,8	2,2	0,84	(0,71 - 0,93)	0,8	2,2
		Hip	0,87	(0,76 - 0,94)	1,7	4,8	0,89	(0,79 - 0,95)	3,2	8,8	0,90	(0,82 - 0,96)	1,6	4,3	0,94	(0,88 - 0,97)	1,2	3,3	0,91	(0,83 - 0,96)	1,5	4,1	0,92	(0,85 - 0,97)	1,3	3,7
		Knee	0,89	(0,79 - 0,95)	1,8	4,9	0,96	(0,92 - 0,98)	1,1	3,1	0,91	(0,84 - 0,96)	1,3	3,7	0,90	(0,82 - 0,96)	0,9	2,5	0,97	(0,94 - 0,99)	1,0	2,7	0,93	(0,87 - 0,97)	0,8	2,3
		Ankle	0,83	(0,7 - 0,92)	1,8	5,0	0,92	(0,86 - 0,97)	1,5	4,1	0,90	(0,8 - 0,95)	1,9	5,2	0,85	(0,73 - 0,93)	2,1	5,7	0,94	(0,88 - 0,97)	1,7	4,6	0,78	(0,62 - 0,9)	2,2	6,1
	transverse	Trunk	0,92	(0,83 - 0,97)	0,2	0,6	0,86	(0,72 - 0,94)	0,3	0,8	0,85	(0,7 - 0,94)	0,3	0,7	0,92	(0,83 - 0,97)	0,2	0,6	0,84	(0,64 - 0,93)	0,3	0,7	0,87	(0,72 - 0,95)	0,2	0,7
		Pelvis	0,92	(0,81 - 0,97)	0,7	1,9	0,86	(0,73 - 0,94)	0,9	2,4	0,86	(0,72 - 0,94)	0,8	2,2	0,92	(0,84 - 0,97)	0,7	1,9	0,83	(0,64 - 0,93)	0,8	2,2	0,87	(0,7 - 0,95)	0,7	2,0
		Hip	0,92	(0,85 - 0,96)	1,9	5,3	0,94	(0,89 - 0,98)	1,8	4,9	0,91	(0,84 - 0,96)	2,3	6,5	0,86	(0,74 - 0,93)	2,0	5,7	0,87	(0,76 - 0,94)	2,2	6,1	0,88	(0,77 - 0,95)	2,0	5,6
		Knee	0,97	(0,95 - 0,99)	1,3	3,5	0,93	(0,87 - 0,97)	1,6	4,5	0,94	(0,89 - 0,98)	1,7	4,7	0,89	(0,79 - 0,95)	3,1	8,5	0,97	(0,94 - 0,99)	1,2	3,4	0,94	(0,89 - 0,98)	1,8	4,9
		Ankle	0,90	(0,82 - 0,96)	2,0	5,6	0,91	(0,84 - 0,96)	1,4	4,0	0,92	(0,85 - 0,96)	1,9	5,4	0,94	(0,89 - 0,98)	1,9	5,2	0,93	(0,86 - 0,1)	2,0	5,7	0,94	(0,89 - 0,98)	1,9	5,2
	sagittal	Trunk	0,95	(0,9 - 0,98)	0,2	0,6	0,96	(0,93 - 0,98)	0,2	0,6	0,94	(0,89 - 0,97)	0,2	0,6	0,93	(0,87 - 0,97)	0,2	0,5	0,94	(0,88 - 0,97)	0,3	0,7	0,93	(0,86 - 0,97)	0,2	0,5
		Pelvis	0,94	(0,89 - 0,98)	0,6	1,7	0,96	(0,92 - 0,98)	0,6	1,8	0,93	(0,87 - 0,97)	0,7	2,0	0,93	(0,87 - 0,97)	0,5	1,5	0,93	(0,87 - 0,97)	0,8	2,2	0,94	(0,88 - 0,97)	0,6	1,5
		Hip	0,78	(0,62 - 0,9)	2,5	7,0	0,89	(0,8 - 0,95)	2,3	6,4	0,87	(0,76 - 0,94)	2,1	5,9	0,85	(0,72 - 0,93)	3,9	10,8	0,95	(0,91 - 0,98)	2,9	8,0	0,93	(0,86 - 0,97)	2,1	5,9
		Knee	0,91	(0,84 - 0,96)	2,1	5,7	0,83	(0,7 - 0,92)	2,5	6,8	0,86	(0,74 - 0,94)	2,2	6,1	0,83	(0,69 - 0,92)	8,3	23,1	0,95	(0,9 - 0,98)	2,4	6,5	0,90	(0,82 - 0,96)	4,2	11,5
		Ankle	0,89	(0,79 - 0,95)	2,0	5,6	0,73	(0,55 - 0,87)	3,2	8,9	0,79	(0,64 - 0,9)	3,1	8,6	0,94	(0,89 - 0,98)	1,7	4,8	0,97	(0,95 - 0,99)	1,2	3,4	0,94	(0,88 - 0,97)	1,6	4,6
between-session	frontal	Trunk	0,82	(0,57 - 0,93)	0,3	0,9	0,86	(0,64 - 0,94)	0,4	1,0	0,10	(0 - 0,65)	0,5	1,3	0,70	(0,23 - 0,88)	0,4	1,1	0,83	(0,55 - 0,93)	0,4	1,2	0,32	(0 - 0,74)	0,5	1,3
		Pelvis	0,82	(0,55 - 0,93)	1,0	2,8	0,85	(0,63 - 0,94)	1,1	3,0	0,01	(0 - 0,62)	1,5	4,1	0,70	(0,22 - 0,88)	1,2	3,3	0,83	(0,56 - 0,93)	1,3	3,6	0,31	(0 - 0,73)	1,5	4,1
		Hip	0,57	(0 - 0,83)	3,0	8,2	0,77	(0,3 - 0,89)	6,1	17,0	0,56	(0 - 0,83)	3,2	8,9	0,33	(0 - 0,74)	3,6	10,0	0,55	(0 - 0,82)	3,6	10,1	0,53	(0 - 0,82)	3,3	9,3
		Knee	0,77	(0,41 - 0,91)	3,2	8,9	0,83	(0,57 - 0,93)	3,2	8,7	0,59	(0 - 0,84)	3,7	10,2	0,68	(0,2 - 0,88)	2,0	5,7	0,78	(0,42 - 0,91)	3,4	9,5	0,71	(0,28 - 0,89)	2,1	5,8
		Ankle	0,58	(0 - 0,83)	4,8	13,2	0,69	(0,2 - 0,88)	3,4	9,5	0,62	(0,03 - 0,85)	4,2	11,5	0,90	(0,76 - 0,96)	2,1	5,8	0,93	(0,83 - 0,97)	2,6	7,2	0,90	(0,61 - 0,97)	1,5	4,1
	transverse	Trunk	0,58	(0 - 0,84)	0,6	1,8	0,51	(0 - 0,81)	0,6	1,7	0,19	(0 - 0,69)	0,6	1,8	0,49	(0 - 0,8)	0,6	1,7	0,53	(0 - 0,82)	0,6	1,8	0,31	(0 - 0,73)	0,6	1,8
		Pelvis	0,58	(0 - 0,84)	1,9	5,4	0,53	(0 - 0,82)	1,8	5,1	0,21	(0 - 0,7)	1,9	5,4	0,49	(0 - 0,8)	1,9	5,2	0,47	(0 - 0,79)	2,0	5,5	0,23	(0 - 0,7)	2,0	5,5
		Hip	0,00	(0 - 0,47)	7,0	19,4	0,07	(0 - 0,64)	7,0	19,4	0,00	(0 - 0,55)	7,5	20,7	0,00	(0 - 0,41)	6,4	17,7	0,07	(0 - 0,64)	6,7	18,5	0,00	(0 - 0,43)	6,6	18,2
		Knee	0,80	(0,5 - 0,92)	5,0	13,9	0,83	(0,57 - 0,93)	3,9	10,8	0,80	(0,5 - 0,92)	4,8	13,3	0,84	(0,59 - 0,94)	4,6	12,9	0,86	(0,64 - 0,94)	4,2	11,6	0,85	(0,61 - 0,94)	4,3	11,9
		Ankle	0,13	(0 - 0,67)	9,2	25,4	0,20	(0 - 0,69)	8,9	24,7	0,22	(0 - 0,7)	9,0	25,0	0,00	(0 - 0,55)	8,4	23,2	0,23	(0 - 0,7)	8,6	23,8	0,32	(0 - 0,74)	8,1	22,4
	sagittal	Trunk	0,52	(0 - 0,81)	0,7	1,9	0,68	(0,22 - 0,87)	0,8	2,2	0,58	(0 - 0,83)	0,8	2,2	0,46	(0 - 0,79)	0,7	2,1	0,70	(0,27 - 0,88)	0,7	2,1	0,40	(0 - 0,76)	0,8	2,2
		Pelvis	0,52	(0 - 0,81)	2,0	5,6	0,67	(0,19 - 0,87)	2,4	6,6	0,56	(0 - 0,83)	2,4	6,5	0,49	(0 - 0,8)	2,2	6,2	0,69	(0,25 - 0,88)	2,3	6,3	0,42	(0 - 0,77)	2,4	6,6
		Hip	0,77	(0,43 - 0,91)	3,3	9,1	0,72	(0,29 - 0,89)	4,3	12,1	0,72	(0,31 - 0,89)	3,9	10,9	0,76	(0,41 - 0,9)	4,9	13,7	0,92	(0,79 - 0,97)	4,9	13,6	0,80	(0,49 - 0,92)	4,5	12,4
		Knee	0,91	(0,77 - 0,96)	2,8	7,7	0,75	(0,34 - 0,9)	4,3	11,9	0,79	(0,47 - 0,92)	3,1	8,5	0,85	(0,61 - 0,94)	8,0	22,2	0,94	(0,85 - 0,98)	3,6	10,1	0,96	(0,91 - 0,99)	3,5	9,7
		Ankle	0,79	(0,47 - 0,92)	4,1	11,4	0,77	(0,41 - 0,91)	4,2	11,6	0,77	(0,43 - 0,91)	4,3	12,0	0,90	(0,75 - 0,96)	2,9	8,0	0,94	(0,79 - 0,98)	2,3	6,3	0,94	(0,86 - 0,98)	2,3	6,4
between-operator	frontal	Trunk	0,81	(0,51 - 0,93)	0,4	1,0	0,85	(0,62 - 0,95)	0,4	1,1	0,45	(0 - 0,8)	0,5	1,3	0,75	(0,34 - 0,91)	0,4	1,1	0,86	(0,63 - 0,95)	0,4	1,1	0,70	(0,19 - 0,89)	0,4	1,1
		Pelvis	0,81	(0,5 - 0,93)	1,1	3,0	0,84	(0,57 - 0,94)	1,2	3,3	0,42	(0 - 0,79)	1,4	3,9	0,76	(0,36 - 0,91)	1,1	3,2	0,86	(0,64 - 0,95)	1,2	3,2	0,70	(0,18 - 0,89)	1,2	3,3
		Hip	0,77	(0,4 - 0,91)	2,7	7,6	0,81	(0,5 - 0,93)	5,0	13,9	0,75	(0,35 - 0,91)	2,8	7,8	0,38	(0 - 0,77)	3,6	9,9	0,54	(0 - 0,83)	3,8	10,5	0,36	(0 - 0,76)	4,2	11,5
		Knee	0,84	(0,56 - 0,94)	2,8	7,8	0,88	(0,69 - 0,96)	2,7	7,4	0,83	(0,54 - 0,94)	2,5	7,0	0,82	(0,52 - 0,93)	1,6	4,4	0,85	(0,59 - 0,94)	2,7	7,4	0,73	(0,25 - 0,9)	1,9	5,4
		Ankle	0,63	(0 - 0,86)	3,2	8,9	0,80	(0,47 - 0,93)	2,8	7,7	0,77	(0,37 - 0,91)	2,9	8,1	0,82	(0,51 - 0,93)	2,8	7,7	0,91	(0,76 - 0,97)	3,1	8,5	0,87	(0,65 - 0,95)	2,1	5,8
	transverse	Trunk	0,46	(0 - 0,8)	0,7	2,0	0,31	(0 - 0,74)	0,7	2,0	0,09	(0 - 0,67)	0,8	2,2	0,30	(0 - 0,74)	0,8	2,1	0,44	(0 - 0,79)	0,8	2,1	0,00	(0 - 0,62)	0,8	2,4
		Pelvis	0,47	(0 - 0,8)	2,2	6,1	0,32	(0 - 0,75)	2,2	6,2	0,08	(0 - 0,66)	2,4	6,7	0,31	(0 - 0,74)	2,3	6,4	0,37	(0 - 0,77)	2,4	6,6	0,00	(0 - 0,62)	2,5	7,1
		Hip	0,63	(0 - 0,86)	4,9	13,5	0,63	(0 - 0,86)	5,4	14,9	0,59	(0 - 0,85)	5,4	15,1	0,13	(0 - 0,68)	5,6	15,5	0,46	(0 - 0,8)	5,5	15,4	0,38	(0 - 0,77)	5,6	15,6
		Knee	0,74	(0,32 - 0,9)	4,3	11,9	0,64	(0,09 - 0,86)	4,2	11,6	0,61	(0,04 - 0,85)	4,8	13,4	0,73	(0,29 - 0,9)	5,1	14,0	0,73	(0,31 - 0,9)	4,9	13,5	0,76	(0,37 - 0,91)	4,3	12,0
		Ankle	0,63	(0 - 0,86)	4,5	12,5	0,65	(0,1 - 0,87)	3,7	10,4	0,75	(0,33 - 0,91)	4,0	11,0	0,72	(0,23 - 0,9)	4,7	13,0	0,77	(0,38 - 0,92)	4,4	12,1	0,75	(0,31 - 0,91)	4,9	13,7
	sagittal	Trunk	0,36	(0 - 0,76)	0,7	2,1	0,42	(0 - 0,79)	0,9	2,6	0,24	(0 - 0,72)	0,8	2,2	0,58	(0 - 0,84)	0,6	1,6	0,33	(0 - 0,74)	1,0	2,7	0,26	(0 - 0,73)	0,7	1,9
		Pelvis	0,33	(0 - 0,75)	2,2	6,2	0,41	(0 - 0,78)	2,8	7,8	0,25	(0 - 0,73)	2,4	6,6	0,60	(0 - 0,85)	1,7	4,8	0,31	(0 - 0,73)	2,9	8,0	0,25	(0 - 0,73)	2,1	5,8
		Hip	0,46	(0 - 0,8)	4,5	12,5	0,24	(0 - 0,71)	6,8	18,9	0,43	(0 - 0,79)	5,4	15,0	0,58	(0 - 0,84)	6,3	17,5	0,63	(0,05 - 0,86)	9,8	27,2	0,63	(0,03 - 0,86)	6,5	18,0
		Knee	0,93	(0,81 - 0,97)	3,0	8,2	0,88	(0,68 - 0,95)	2,8	7,8	0,89	(0,71 - 0,96)	2,6	7,3	0,85	(0,61 - 0,95)	6,5	18,2	0,89	(0,7 - 0,96)	4,7	13,1	0,97	(0,91 - 0,99)	3,6	9,9
		Ankle	0,82	(0,4 - 0,94)	3,2	8,9	0,87	(0,62 - 0,96)	2,6	7,2	0,84	(0,59 - 0,94)	3,8	10,6	0,92	(0,78 - 0,97)	2,4	6,8	0,92	(0,65 - 0,98)	2,1	5,7	0,96	(0,88 - 0,98)	1,9	5,3

Table S4: Between-session and between-operator reliability (ICC, confidence intervals) and agreement (SEM, MDC) of joint angles from upstairs.

			minSW				minST				maxSW				maxST			
			ICC	CI	SEM	MDC	ICC	CI	SEM	MDC	ICC	CI	SEM	MDC	ICC	CI	SEM	MDC
within-session	frontal	Trunk	0,88	(0,77 - 0,95)	0,2	0,5	0,87	(0,74 - 0,95)	0,2	0,5	0,88	(0,77 - 0,95)	0,3	0,7	0,88	(0,77 - 0,95)	0,2	0,6
		Pelvis	0,92	(0,83 - 0,97)	0,8	2,1	0,85	(0,71 - 0,94)	0,7	2,1	0,88	(0,76 - 0,95)	0,8	2,3	0,82	(0,66 - 0,93)	1,4	3,8
		Hip	0,93	(0,86 - 0,97)	1,6	4,4	0,95	(0,9 - 0,98)	1,3	3,6	0,95	(0,9 - 0,98)	1,1	3,2	0,92	(0,84 - 0,97)	1,6	4,4
		Knee	0,98	(0,96 - 0,99)	1,2	3,4	0,98	(0,96 - 0,99)	1,0	2,8	0,94	(0,88 - 0,98)	0,8	2,3	0,96	(0,92 - 0,99)	0,6	1,6
		Ankle	0,93	(0,85 - 0,97)	2,0	5,5	0,90	(0,8 - 0,96)	2,0	5,6	0,92	(0,83 - 0,97)	2,2	6,0	0,89	(0,78 - 0,96)	1,7	4,8
	transverse	Trunk	0,94	(0,87 - 0,98)	0,2	0,4	0,93	(0,85 - 0,98)	0,2	0,4	0,91	(0,82 - 0,97)	0,2	0,6	0,91	(0,8 - 0,96)	0,2	0,5
		Pelvis	0,95	(0,89 - 0,98)	0,5	1,3	0,93	(0,85 - 0,97)	0,5	1,4	0,91	(0,81 - 0,96)	0,6	1,8	0,92	(0,84 - 0,97)	0,6	1,7
		Hip	0,90	(0,78 - 0,96)	1,8	4,9	0,92	(0,83 - 0,97)	1,8	5,0	0,90	(0,81 - 0,96)	1,5	4,1	0,91	(0,82 - 0,97)	1,4	3,8
		Knee	0,92	(0,84 - 0,97)	1,6	4,5	0,97	(0,93 - 0,99)	1,1	3,0	0,97	(0,94 - 0,99)	1,3	3,6	0,97	(0,93 - 0,99)	1,1	3,1
		Ankle	0,96	(0,91 - 0,98)	1,7	4,6	0,95	(0,89 - 0,98)	1,9	5,3	0,93	(0,85 - 0,97)	2,1	5,7	0,92	(0,84 - 0,97)	2,3	6,4
	sagittal	Trunk	0,96	(0,91 - 0,98)	0,1	0,4	0,94	(0,88 - 0,98)	0,2	0,4	0,97	(0,94 - 0,99)	0,2	0,5	0,98	(0,96 - 0,99)	0,1	0,4
		Pelvis	0,95	(0,89 - 0,98)	0,4	1,2	0,94	(0,87 - 0,98)	0,5	1,4	0,97	(0,94 - 0,99)	0,5	1,3	0,92	(0,85 - 0,97)	0,9	2,6
		Hip	0,93	(0,86 - 0,97)	1,5	4,2	0,94	(0,88 - 0,98)	1,3	3,7	0,97	(0,95 - 0,99)	1,2	3,5	0,98	(0,96 - 0,99)	1,2	3,3
		Knee	0,91	(0,82 - 0,97)	2,2	6,1	0,96	(0,91 - 0,98)	1,4	3,9	0,96	(0,91 - 0,98)	1,7	4,6	0,96	(0,93 - 0,99)	1,3	3,7
		Ankle	0,69	(0,47 - 0,86)	2,9	8,2	0,55	(0,31 - 0,79)	3,3	9,1	0,96	(0,92 - 0,99)	1,4	3,8	0,96	(0,92 - 0,99)	1,1	3,0
between-session	frontal	Trunk	0,60	(0,05 - 0,84)	1,6	4,5	0,60	(0,05 - 0,84)	0,5	1,3	0,69	(0,2 - 0,88)	0,4	1,1	0,47	(0 - 0,79)	0,4	1,0
		Pelvis	0,67	(0,18 - 0,87)	3,4	9,5	0,58	(0 - 0,83)	1,7	4,6	0,67	(0,14 - 0,87)	1,3	3,5	0,14	(0 - 0,66)	2,0	5,5
		Hip	0,78	(0,44 - 0,91)	6,0	16,7	0,78	(0,42 - 0,91)	3,3	9,3	0,62	(0,03 - 0,85)	3,3	9,0	0,68	(0,19 - 0,87)	3,4	9,4
		Knee	0,68	(0,21 - 0,87)	6,5	17,9	0,68	(0,17 - 0,87)	5,1	14,2	0,40	(0 - 0,77)	4,1	11,3	0,27	(0 - 0,71)	2,9	8,2
		Ankle	0,41	(0 - 0,79)	0,7	1,9	0,39	(0 - 0,76)	5,6	15,4	0,59	(0 - 0,84)	5,0	13,9	0,82	(0,56 - 0,93)	2,5	6,9
	transverse	Trunk	0,20	(0 - 0,69)	0,7	1,9	0,18	(0 - 0,68)	0,7	1,9	0,00	(0 - 0,57)	0,7	1,9	0,28	(0 - 0,71)	0,6	1,7
		Pelvis	0,20	(0 - 0,69)	2,0	5,6	0,25	(0 - 0,71)	1,9	5,2	0,03	(0 - 0,62)	2,0	5,6	0,30	(0 - 0,72)	2,0	5,4
		Hip	0,14	(0 - 0,66)	5,9	16,4	0,00	(0 - 0,53)	6,4	17,7	0,00	(0 - 0,53)	6,1	16,9	0,00	(0 - 0,58)	5,9	16,5
		Knee	0,72	(0,32 - 0,89)	4,1	11,4	0,60	(0,06 - 0,84)	5,2	14,4	0,85	(0,62 - 0,94)	4,4	12,1	0,65	(0,15 - 0,86)	4,8	13,3
		Ankle	0,52	(0 - 0,81)	9,3	25,7	0,10	(0 - 0,65)	12,6	34,9	0,62	(0,01 - 0,85)	7,6	21,1	0,44	(0 - 0,78)	10,2	28,2
	sagittal	Trunk	0,00	(0 - 0,6)	0,8	2,2	0,15	(0 - 0,67)	0,7	2,0	0,05	(0 - 0,63)	0,9	2,4	0,13	(0 - 0,66)	0,9	2,5
		Pelvis	0,06	(0 - 0,64)	2,3	6,5	0,27	(0 - 0,71)	2,1	5,7	0,10	(0 - 0,65)	2,5	7,1	0,13	(0 - 0,66)	2,7	7,5
		Hip	0,06	(0 - 0,63)	6,3	17,4	0,00	(0 - 0,51)	6,7	18,5	0,21	(0 - 0,69)	7,6	21,1	0,24	(0 - 0,7)	7,8	21,6
		Knee	0,92	(0,8 - 0,97)	2,6	7,1	0,91	(0,77 - 0,97)	2,5	6,8	0,84	(0,4 - 0,95)	3,7	10,4	0,86	(0,42 - 0,95)	3,1	8,7
		Ankle	0,82	(0,55 - 0,93)	4,0	11,0	0,78	(0,45 - 0,91)	4,0	11,0	0,89	(0,73 - 0,96)	3,2	9,0	0,89	(0,72 - 0,96)	2,3	6,4
between-operator	frontal	Trunk	0,48	(0 - 0,79)	0,6	1,7	0,47	(0 - 0,79)	0,6	1,8	0,69	(0,21 - 0,88)	0,5	1,4	0,40	(0 - 0,77)	0,5	1,5
		Pelvis	0,57	(0 - 0,83)	2,0	5,6	0,57	(0 - 0,83)	1,8	5,0	0,71	(0,24 - 0,88)	1,5	4,2	0,33	(0 - 0,74)	2,3	6,5
		Hip	0,64	(0,07 - 0,86)	4,0	11,1	0,81	(0,5 - 0,92)	3,4	9,5	0,61	(0 - 0,85)	4,1	11,5	0,68	(0,19 - 0,87)	4,3	11,8
		Knee	0,69	(0,2 - 0,88)	5,2	14,5	0,70	(0,26 - 0,88)	4,4	12,2	0,47	(0 - 0,79)	3,6	10,0	0,53	(0 - 0,82)	2,8	7,8
		Ankle	0,09	(0 - 0,64)	7,5	20,7	0,14	(0 - 0,67)	5,7	15,7	0,64	(0,15 - 0,86)	4,0	11,1	0,89	(0,73 - 0,96)	1,9	5,2
	transverse	Trunk	0,00	(0 - 0,58)	0,9	2,4	0,00	(0 - 0,59)	0,8	2,3	0,00	(0 - 0,09)	0,9	2,4	0,00	(0 - 0,37)	0,9	2,4
		Pelvis	0,00	(0 - 0,48)	2,7	7,4	0,00	(0 - 0,52)	2,4	6,8	0,00	(0 - 0,17)	2,7	7,5	0,00	(0 - 0,49)	2,6	7,2
		Hip	0,32	(0 - 0,73)	5,4	14,9	0,49	(0 - 0,8)	5,3	14,7	0,52	(0 - 0,81)	4,6	12,6	0,52	(0 - 0,81)	4,7	12,9
		Knee	0,36	(0 - 0,75)	5,5	15,3	0,42	(0 - 0,77)	5,4	15,0	0,56	(0 - 0,83)	6,1	17,0	0,62	(0,03 - 0,85)	5,1	14,1
		Ankle	0,08	(0 - 0,64)	11,4	31,7	0,00	(0 - 0,41)	13,5	37,5	0,30	(0 - 0,72)	9,4	26,0	0,05	(0 - 0,61)	12,1	33,6
	sagittal	Trunk	0,28	(0 - 0,71)	1,1	2,9	0,33	(0 - 0,73)	1,0	2,9	0,25	(0 - 0,7)	1,1	3,0	0,27	(0 - 0,71)	1,1	3,0
		Pelvis	0,28	(0 - 0,72)	3,1	8,7	0,34	(0 - 0,74)	3,1	8,5	0,18	(0 - 0,68)	3,3	9,3	0,13	(0 - 0,65)	3,4	9,4
		Hip	0,33	(0 - 0,74)	7,0	19,3	0,31	(0 - 0,73)	7,0	19,5	0,51	(0 - 0,81)	7,7	21,4	0,42	(0 - 0,77)	8,0	22,2
		Knee	0,87	(0,67 - 0,95)	3,0	8,3	0,88	(0,7 - 0,95)	3,0	8,3	0,88	(0,7 - 0,95)	3,9	10,8	0,80	(0,5 - 0,92)	4,5	12,4
		Ankle	0,77	(0,43 - 0,91)	4,9	13,7	0,71	(0,3 - 0,88)	4,3	12,0	0,89	(0,71 - 0,96)	3,2	8,9	0,89	(0,72 - 0,96)	2,1	5,7

Table S5: Between-session and between-operator reliability (ICC, confidence intervals) and agreement (SEM, MDC) of joint angles from downstairs.

			minSW				minST				maxSW				maxST			
			ICC	CI	SEM	MDC	ICC	CI	SEM	MDC	ICC	CI	SEM	MDC	ICC	CI	SEM	MDC
within-session	frontal	Trunk	0,93	(0,86 - 0,97)	0,2	0,5	0,93	(0,87 - 0,97)	0,2	0,5	0,91	(0,83 - 0,96)	0,2	0,6	0,90	(0,81 - 0,96)	0,2	0,5
		Pelvis	0,80	(0,64 - 0,91)	0,9	2,4	0,93	(0,86 - 0,97)	0,7	1,9	0,88	(0,78 - 0,95)	0,7	2,0	0,74	(0,56 - 0,88)	0,9	2,5
		Hip	0,91	(0,82 - 0,96)	1,8	5,1	0,92	(0,84 - 0,97)	1,4	3,9	0,90	(0,81 - 0,96)	1,6	4,4	0,87	(0,76 - 0,94)	1,8	5,1
		Knee	0,93	(0,86 - 0,97)	1,6	4,4	0,92	(0,84 - 0,97)	1,2	3,3	0,95	(0,9 - 0,98)	0,9	2,5	0,96	(0,91 - 0,98)	0,8	2,3
		Ankle	0,94	(0,88 - 0,97)	1,5	4,2	0,92	(0,85 - 0,97)	1,6	4,4	0,92	(0,85 - 0,97)	1,9	5,3	0,85	(0,72 - 0,93)	1,7	4,8
	transverse	Trunk	0,90	(0,8 - 0,96)	0,2	0,5	0,83	(0,7 - 0,92)	0,3	0,7	0,89	(0,79 - 0,95)	0,2	0,6	0,89	(0,79 - 0,95)	0,2	0,5
		Pelvis	0,74	(0,56 - 0,88)	1,0	2,8	0,77	(0,61 - 0,89)	1,1	3,0	0,87	(0,75 - 0,94)	0,7	1,8	0,76	(0,59 - 0,89)	1,0	2,8
		Hip	0,87	(0,75 - 0,94)	2,1	5,9	0,91	(0,82 - 3,7)	1,9	5,3	0,92	(0,84 - 0,96)	1,8	4,9	0,85	(0,7 - 0,94)	2,0	5,5
		Knee	0,93	(0,87 - 0,97)	1,9	5,3	0,92	(0,86 - 0,97)	2,0	5,5	0,96	(0,93 - 0,98)	1,2	3,4	0,96	(0,91 - 0,98)	1,3	3,6
		Ankle	0,94	(0,89 - 0,98)	2,1	5,8	0,95	(0,9 - 0,98)	2,3	6,4	0,96	(0,92 - 0,98)	2,0	5,4	0,96	(0,92 - 0,98)	2,0	5,7
	sagittal	Trunk	0,92	(0,84 - 0,96)	0,2	0,5	0,87	(0,75 - 0,94)	0,2	0,7	0,91	(0,82 - 0,96)	0,2	0,6	0,93	(0,87 - 0,97)	0,2	0,5
		Pelvis	0,83	(0,69 - 0,92)	0,9	2,4	0,92	(0,84 - 0,97)	0,7	1,9	0,88	(0,78 - 0,95)	0,7	1,9	0,87	(0,76 - 0,94)	0,9	2,4
		Hip	0,91	(0,83 - 0,96)	1,7	4,7	0,85	(0,72 - 0,93)	2,3	6,3	0,92	(0,84 - 0,97)	2,1	5,7	0,91	(0,83 - 0,96)	1,8	4,9
		Knee	0,93	(0,86 - 0,97)	1,6	4,4	0,93	(0,86 - 0,97)	1,3	3,7	0,92	(0,84 - 0,96)	2,1	5,9	0,63	(0,42 - 0,82)	3,5	9,8
		Ankle	0,87	(0,75 - 0,94)	1,8	4,9	0,78	(0,62 - 0,9)	2,8	7,8	0,92	(0,84 - 0,96)	2,9	8,1	0,96	(0,92 - 0,98)	1,7	4,6
between-session	frontal	Trunk	0,68	(0,24 - 0,87)	0,4	1,1	0,68	(0,24 - 0,87)	0,4	1,2	0,75	(0,35 - 0,9)	0,4	1,0	0,67	(0,16 - 0,87)	0,4	1,1
		Pelvis	0,70	(0,25 - 0,88)	1,1	3,2	0,47	(0 - 0,79)	1,8	5,0	0,57	(0 - 0,83)	1,3	3,5	0,40	(0 - 0,75)	1,3	3,7
		Hip	0,71	(0,26 - 0,89)	3,4	9,5	0,79	(0,48 - 0,92)	0,9	2,4	0,75	(0,37 - 0,9)	2,8	7,9	0,72	(0,29 - 0,89)	3,1	8,5
		Knee	0,69	(0,23 - 0,88)	5,3	14,6	0,56	(0 - 0,82)	4,7	12,9	0,24	(0 - 0,7)	3,9	10,8	0,23	(0 - 0,7)	3,6	10,1
		Ankle	0,59	(0 - 0,83)	4,6	12,8	0,58	(0 - 0,83)	4,3	11,8	0,53	(0 - 0,82)	5,8	16,2	0,84	(0,57 - 0,94)	2,4	6,7
	transverse	Trunk	0,00	(0 - 0,58)	0,6	1,6	0,16	(0 - 0,67)	0,6	1,6	0,15	(0 - 0,67)	0,6	1,6	0,30	(0 - 0,72)	0,5	1,5
		Pelvis	0,25	(0 - 0,71)	1,9	5,4	0,24	(0 - 0,7)	2,0	5,7	0,22	(0 - 0,69)	1,8	4,9	0,00	(0 - 0,53)	1,9	5,2
		Hip	0,00	(0 - 0,55)	6,1	16,8	0,01	(0 - 0,62)	5,8	16,0	0,02	(0 - 0,63)	5,9	16,3	0,00	(0 - 0,51)	0,9	2,5
		Knee	0,76	(0,4 - 0,9)	4,7	13,1	0,65	(0,16 - 0,86)	4,8	13,3	0,79	(0,49 - 0,92)	3,9	10,7	0,64	(0,14 - 0,86)	4,2	11,7
		Ankle	0,51	(0 - 0,81)	8,3	22,9	0,26	(0 - 0,71)	10,7	29,7	0,56	(0 - 0,83)	8,5	23,5	0,57	(0 - 0,83)	8,9	24,6
	sagittal	Trunk	0,18	(0 - 0,68)	0,8	2,3	0,11	(0 - 0,66)	0,8	2,3	0,18	(0 - 0,68)	0,9	2,4	0,24	(0 - 0,71)	0,9	2,6
		Pelvis	0,19	(0 - 0,69)	2,7	7,4	0,26	(0 - 0,71)	3,0	8,3	0,27	(0 - 0,72)	2,4	6,7	0,22	(0 - 0,7)	2,7	7,5
		Hip	0,22	(0 - 0,69)	6,6	18,3	0,00	(0 - 0,58)	6,9	19,0	0,46	(0 - 0,78)	6,6	18,4	0,14	(0 - 0,66)	6,7	18,6
		Knee	0,90	(0,62 - 0,97)	2,2	6,2	0,89	(0,6 - 0,96)	2,2	6,0	0,87	(0,36 - 0,96)	2,8	7,7	0,74	(0,38 - 0,9)	3,9	10,9
		Ankle	0,80	(0,47 - 0,92)	3,5	9,8	0,78	(0,43 - 0,91)	4,0	11,1	0,96	(0,9 - 0,99)	2,5	6,9	0,96	(0,91 - 0,99)	2,3	6,4
between-operator	frontal	Trunk	0,70	(0,27 - 0,88)	0,5	1,4	0,70	(0,27 - 0,88)	0,5	1,5	0,71	(0,26 - 0,89)	0,5	1,4	0,65	(0,11 - 0,86)	0,5	1,4
		Pelvis	0,72	(0,3 - 0,89)	1,5	4,0	0,48	(0 - 0,8)	2,1	5,9	0,67	(0,15 - 0,87)	1,6	4,4	0,50	(0 - 0,8)	1,5	4,1
		Hip	0,79	(0,47 - 0,92)	3,7	10,3	0,79	(0,47 - 0,92)	3,8	10,4	0,75	(0,34 - 0,9)	3,7	10,3	0,78	(0,43 - 0,91)	3,6	10,1
		Knee	0,77	(0,4 - 0,91)	4,2	11,7	0,76	(0,39 - 0,91)	3,3	9,2	0,35	(0 - 0,74)	3,5	9,8	0,62	(0,02 - 0,85)	3,2	9,0
		Ankle	0,09	(0 - 0,62)	6,3	17,5	0,17	(0 - 0,66)	6,9	19,2	0,21	(0 - 0,68)	7,2	19,8	0,77	(0,44 - 0,91)	2,6	7,3
	transverse	Trunk	0,01	(0 - 0,61)	0,7	2,0	0,00	(0 - 0,52)	0,8	2,2	0,11	(0 - 0,65)	0,7	1,9	0,05	(0 - 0,62)	0,7	2,0
		Pelvis	0,33	(0 - 0,74)	2,2	6,1	0,31	(0 - 0,73)	2,4	6,8	0,21	(0 - 0,68)	2,0	5,6	0,19	(0 - 0,67)	2,5	6,9
		Hip	0,48	(0 - 0,8)	4,2	11,5	0,64	(0,05 - 0,86)	3,9	10,8	0,43	(0 - 0,78)	4,3	12,0	0,20	(0 - 0,69)	4,5	12,6
		Knee	0,63	(0,08 - 0,85)	5,0	13,9	0,57	(0 - 0,83)	5,1	14,0	0,50	(0 - 0,81)	5,8	16,0	0,55	(0 - 0,82)	4,8	13,4
		Ankle	0,00	(0 - 0,41)	11,4	31,6	0,00	(0 - 0,33)	12,8	35,4	0,08	(0 - 0,62)	10,5	29,1	0,00	(0 - 0,54)	11,8	32,7
	sagittal	Trunk	0,41	(0 - 0,77)	1,0	2,9	0,35	(0 - 0,74)	1,1	3,1	0,36	(0 - 0,75)	1,0	2,8	0,36	(0 - 0,75)	1,1	2,9
		Pelvis	0,35	(0 - 0,74)	3,3	9,2	0,33	(0 - 0,74)	3,6	10,1	0,31	(0 - 0,73)	3,1	8,5	0,36	(0 - 0,75)	3,1	8,6
		Hip	0,11	(0 - 0,66)	6,7	18,5	0,08	(0 - 0,65)	6,7	18,6	0,53	(0 - 0,82)	7,2	20,1	0,00	(0 - 0,59)	7,0	19,4
		Knee	0,81	(0,52 - 0,92)	3,8	10,4	0,76	(0,42 - 0,91)	3,9	10,7	0,83	(0,58 - 0,93)	3,9	10,8	0,79	(0,47 - 0,91)	3,6	9,8
		Ankle	0,70	(0,27 - 0,88)	4,9	13,5	0,57	(0 - 0,83)	4,6	12,9	0,91	(0,77 - 0,96)	4,2	11,6	0,95	(0,88 - 0,98)	2,6	7,2

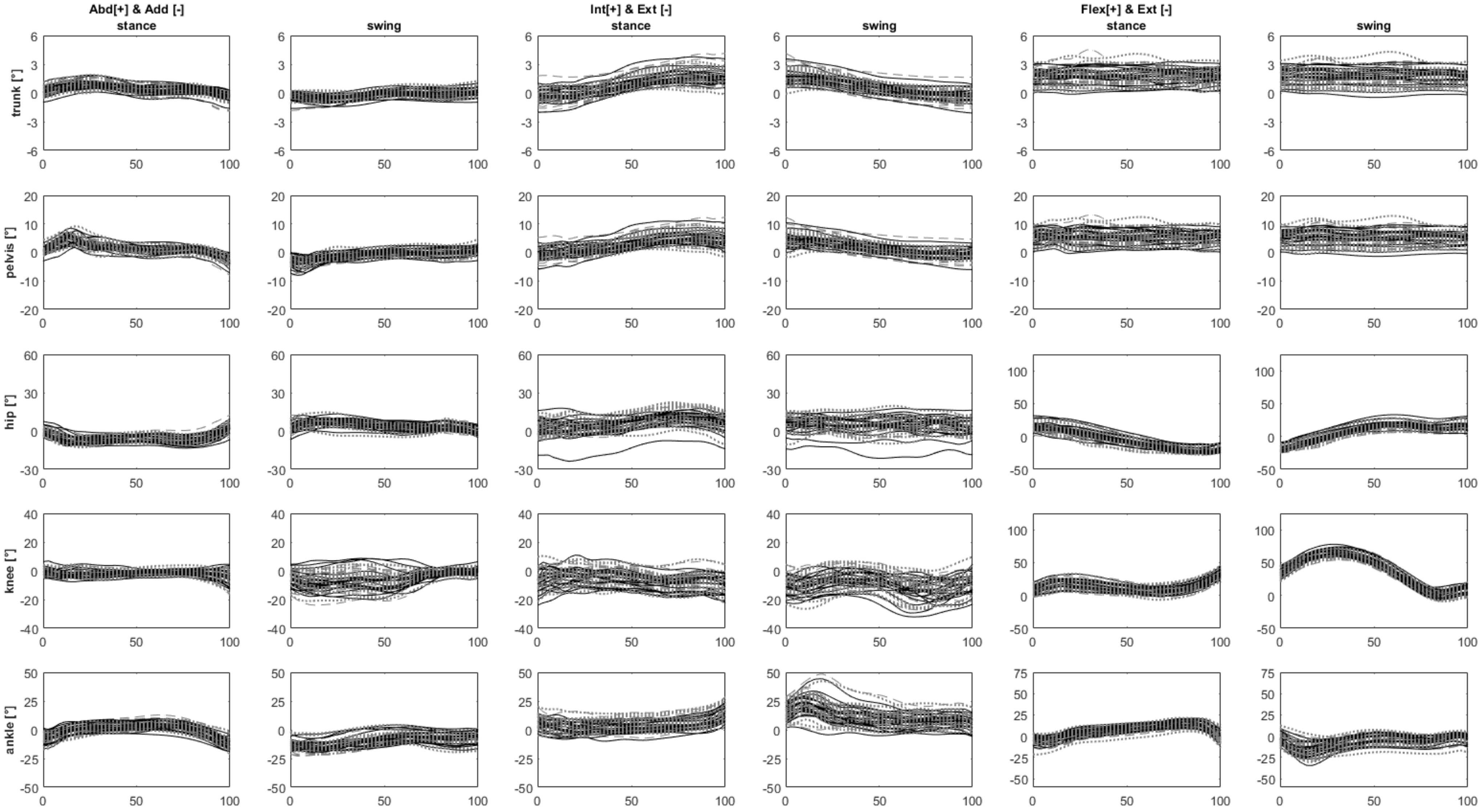

Figure S6: Waveforms of the walking task of all participants (n=20) from the trunk, pelvis, hip, knee and ankle, abd-adduction, int-external rotation and flexion-extension angles during swing and stance phases, from the three sessions: day1-operator1 (solid); day1-operator2 (striped); day2-operator1 (dotted).

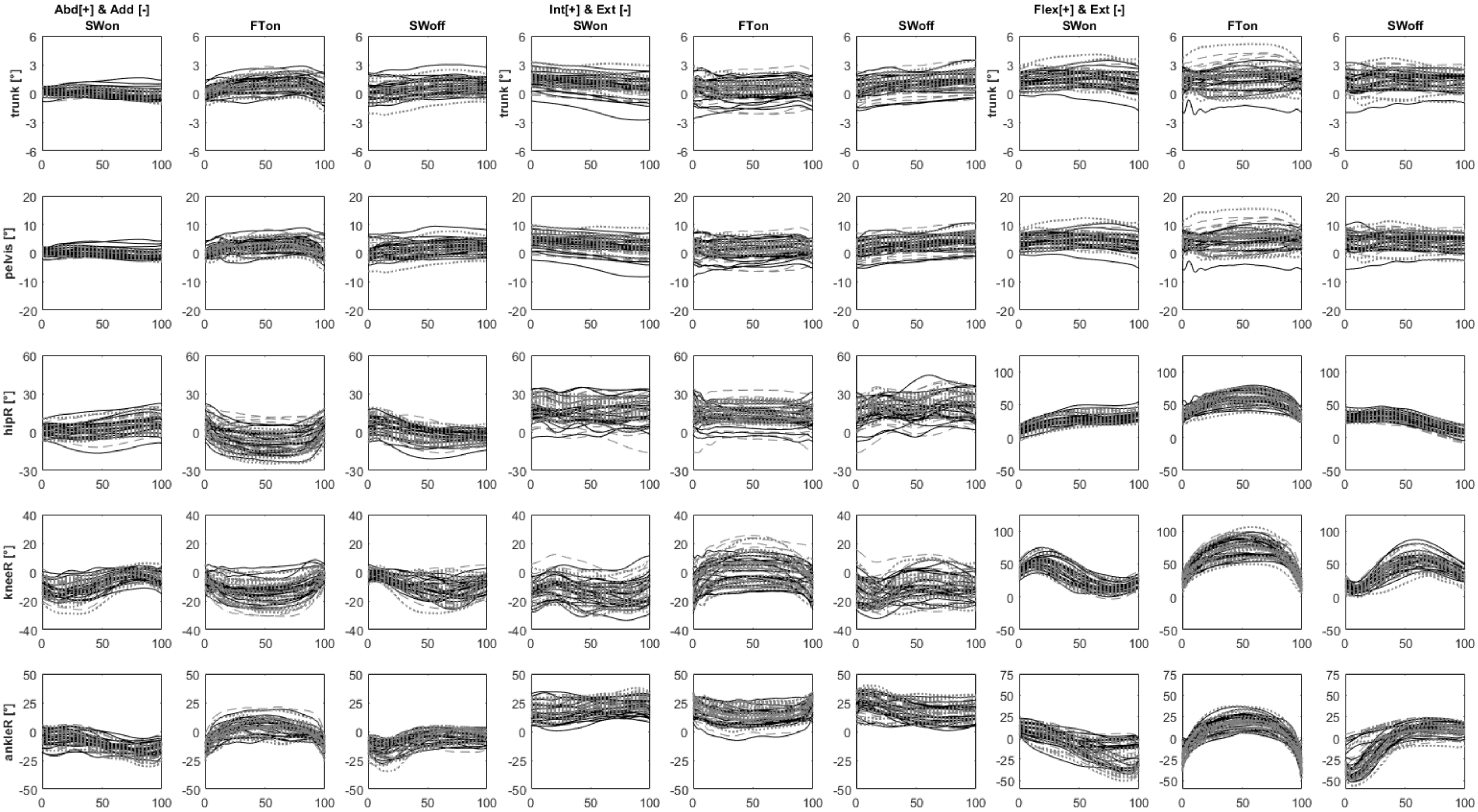

Figure S7: Waveforms of the forward lunge task of all participants (n=20) from the trunk, pelvis, hip, knee and ankle, abd-adduction, int-external rotation and flexion-extension angles during the swing towards foot contact (SWon), foot contact on the ground (FTon) and swing backwards (SWoff), from the three sessions: day1-operator1 (solid); day1-operator2 (striped); day2-operator1 (dotted).

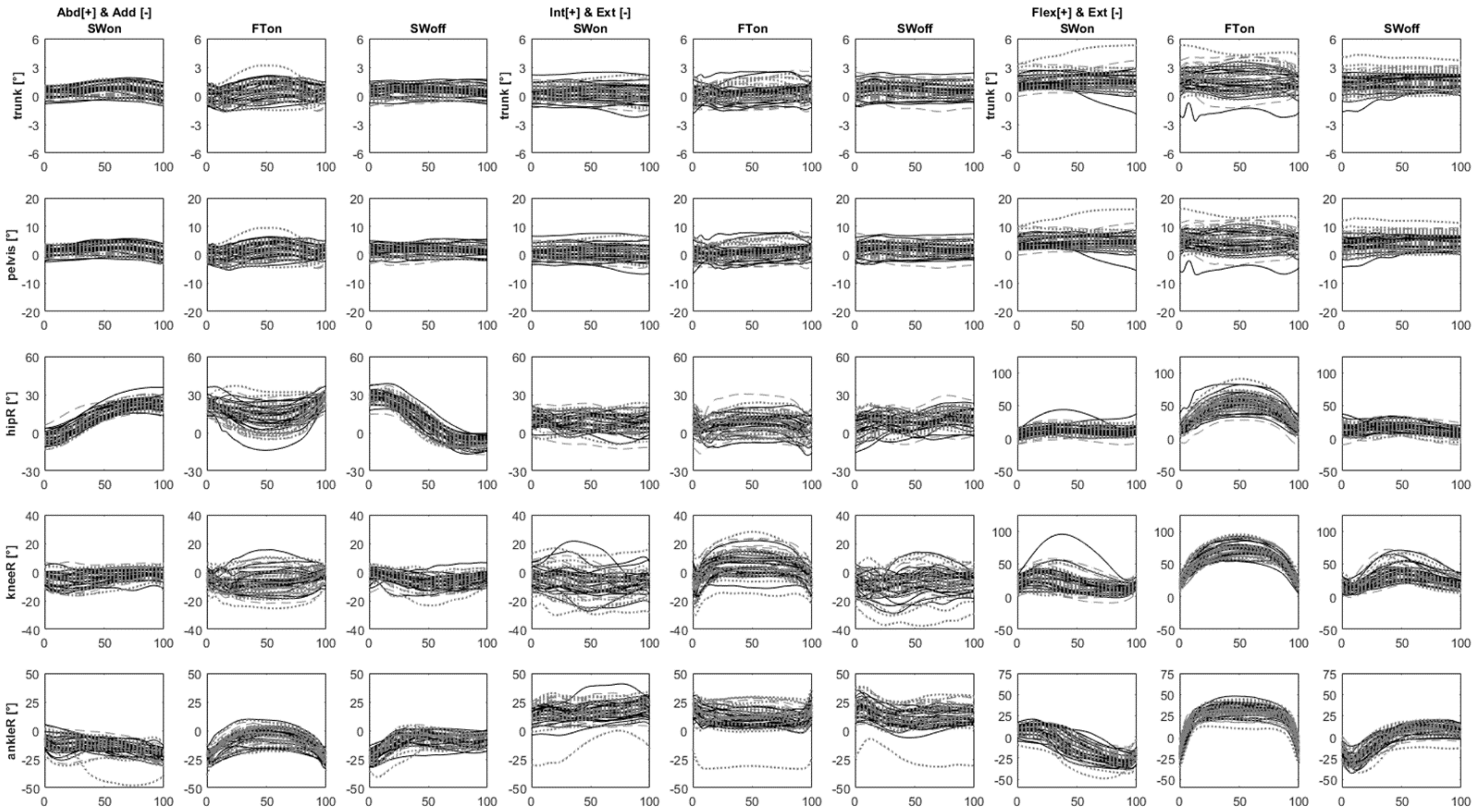

Figure S8: Waveforms of the sideward lunge task of all participants (n=20) from the trunk, pelvis, hip, knee and ankle, abd-adduction, int-external rotation and flexion-extension angles during the swing towards foot contact (SWon), foot contact on the ground (FTon) and swing backwards (SWoff), from the three sessions: day1-operator1 (solid); day1-operator2 (striped); day2-operator1 (dotted).

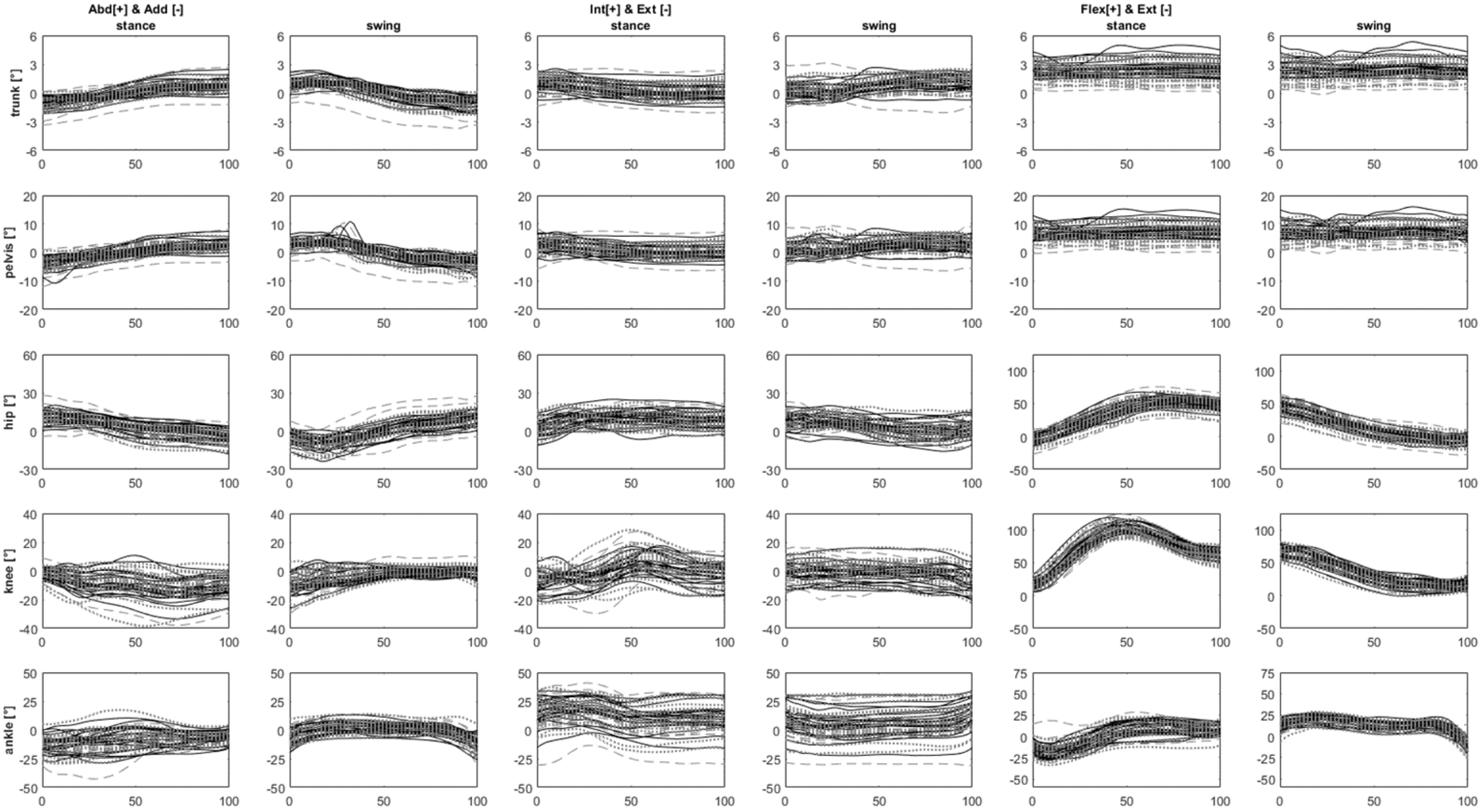

Figure S9: Waveforms of the upstairs task of all participants (n=20) from the trunk, pelvis, hip, knee and ankle, abd-adduction, int-external rotation and flexion-extension angles during swing and stance phases, from the three sessions: day1-operator1 (solid); day1-operator2 (striped); day2-operator1 (dotted).

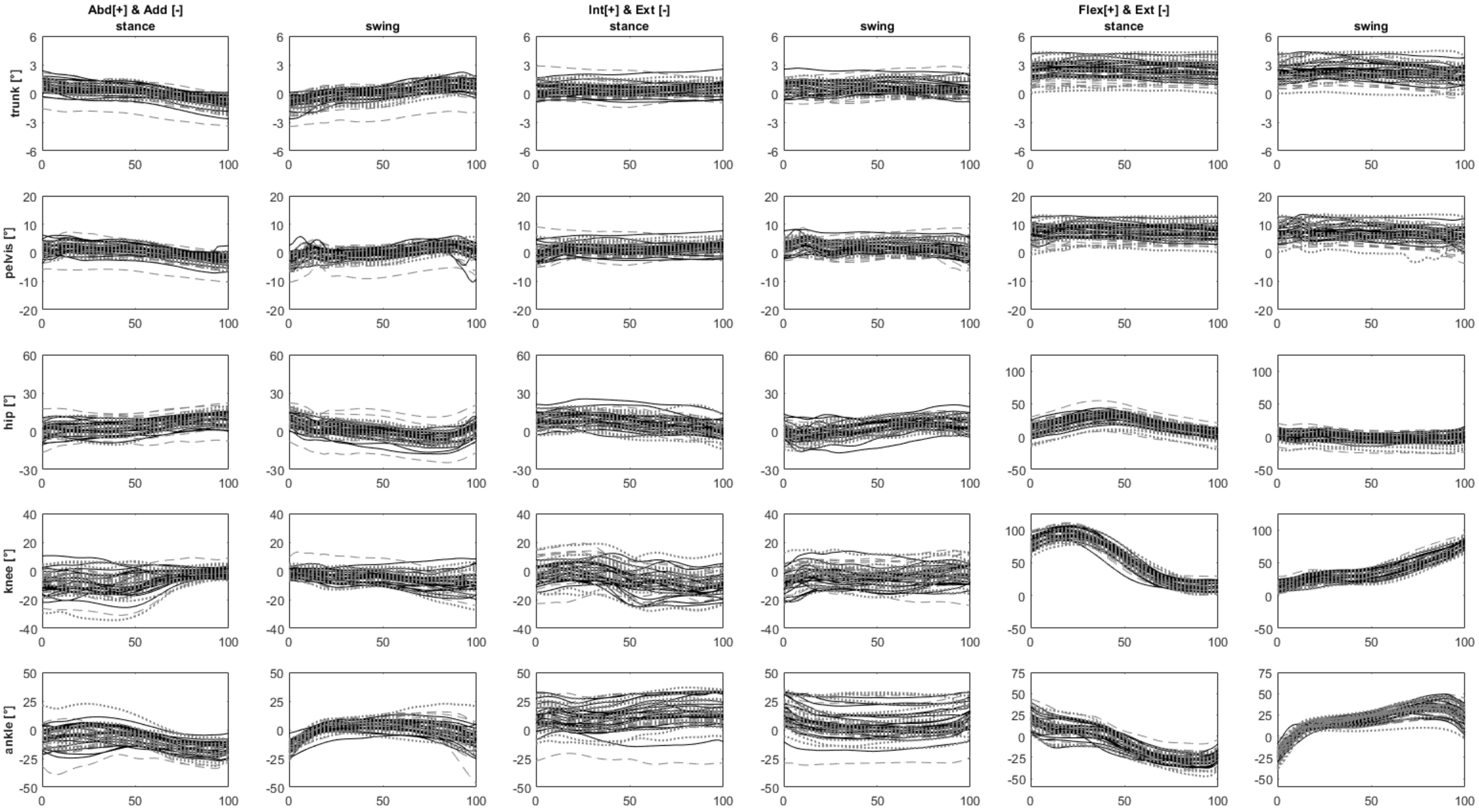

Figure S10: Waveforms of the downstairs task of all participants (n=20) from the trunk, pelvis, hip, knee and ankle, abd-adduction, int-external rotation and flexion-extension angles during swing and stance phases, from the three sessions: day1-operator1 (solid); day1-operator2 (striped); day2-operator1 (dotted).

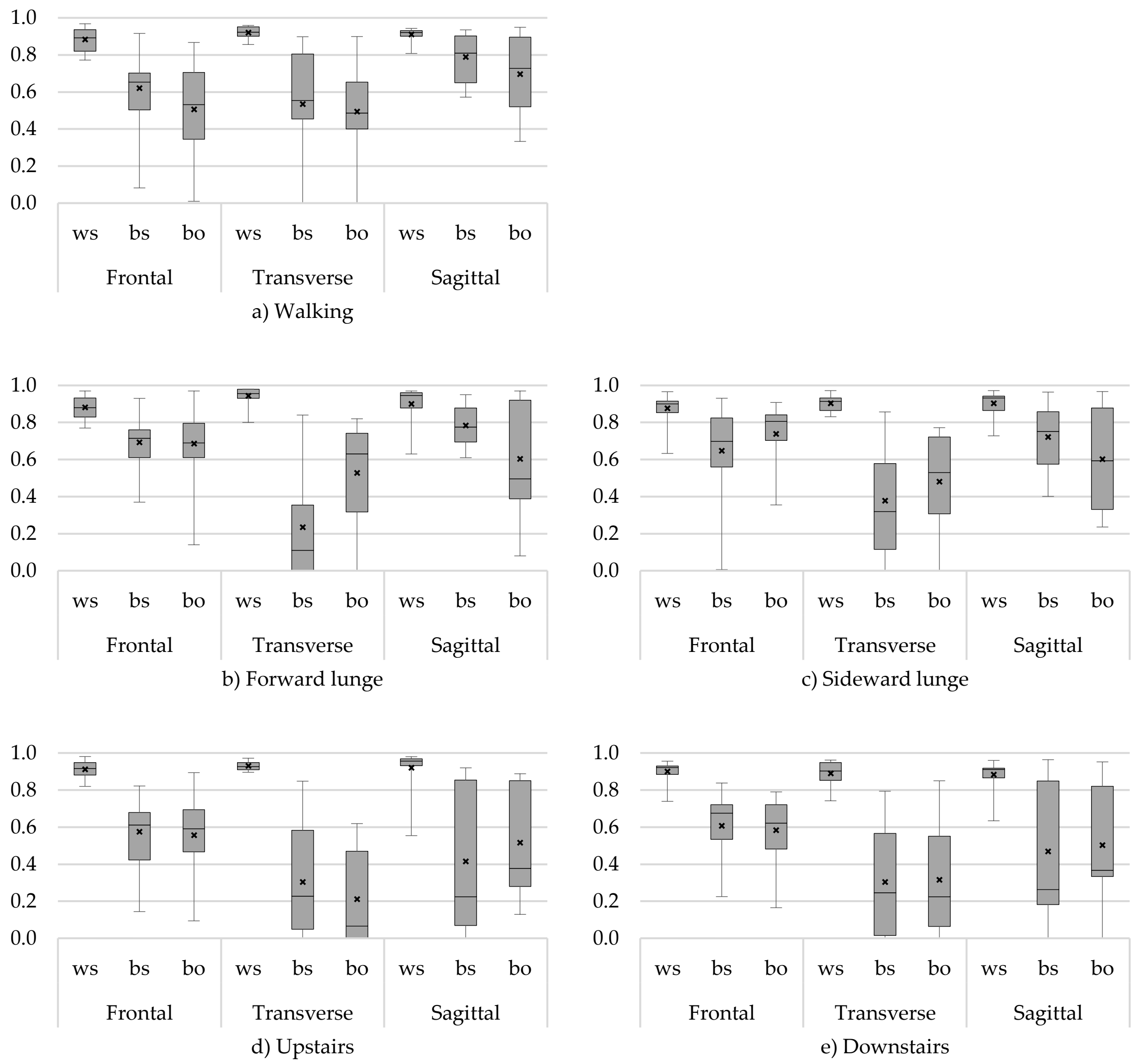

Figure S11: Comparison of the thee levels of reliability from all angles and movement planes of (**a**) Walking, (**b**) Forward lunge, (**c**) Sideward lunge, (**d**) Upstairs and (**e**) Downstairs. With on the x-axis the within-session (ws), between-session (bs) and between-operator (bo) reliability in the frontal, transverse and sagittal plane and on the y-axis the ICC.
